# Impact of Processing Method and Storage Time on Phytochemical Concentrations in an Antioxidant-Rich Food Mixture

**DOI:** 10.3390/antiox12061252

**Published:** 2023-06-10

**Authors:** Julia N. DeBenedictis, Theo M. de Kok, Simone G. van Breda

**Affiliations:** Maastricht University Medical Center, Department of Toxicogenomics, GROW—School for Oncology and Reproduction, P.O. Box 616, 6200 MD Maastricht, The Netherlands

**Keywords:** antioxidants, food processing, pascalization, phytochemicals, methods

## Abstract

Foods high in phytochemicals are known for their role in the prevention of chronic disease development, but after processing and storage, such food products may lose part of their functionality as these compounds are sensitive to the impact of processing temperature and the type of methods applied. Therefore, we measured the levels of vitamin C, anthocyanins, carotenoids, catechins, chlorogenic acid, and sulforaphane in a complex blend of fruits and vegetables, and when applied to a dry food product, after exposure to different processing methods. These levels were compared between pasteurized, pascalized (high-pressure processing), and untreated conditions. Furthermore, we established the effect of freezing and storage time on the stability of these compounds. The results showed that pascalization better preserved vitamin C and sulforaphane, whereas pasteurization resulted in higher concentrations of chlorogenic acid, carotenoids, and catechins. For samples which were frozen and thawed immediately after processing, pascalization was the optimal treatment for higher contents of lutein, cyanidin-3-glucoside, quercetin-3-glucoside, delphinidin-3-glucoside, peonidin-3-glucoside, and epicatechin gallate. Ultimately, the optimal processing method to preserve phytochemicals in fruit and vegetable products is as complex as the blend of compounds, and this decision-making would best be led by the prioritized nutrient aim of an antioxidant food product.

## 1. Introduction

The health-benefits of fruits and vegetables are well established and due in large part to their phytochemical content. Phytochemicals are secondary plant metabolites which exert antioxidant effects in the human body [1]. Antioxidant-rich foods help mitigate the deleterious effects of reactive oxygen species in the body, reducing oxidative stress and subsequent cellular damage, inflammation, and other oxidative reactions related to DNA, proteins, and cellular lipids. In turn, the daily consumption of fruits and vegetables has been linked to a reduced risk of cardiovascular disease, cancer, poor cognitive performance, and other diet-related diseases [2]. However, traditional high-heat processing methods of antioxidant-rich foods are known to reduce the content of some phytochemicals, including antioxidants, diminishing their expected health effects [3].

The conventional method for commercial food preservation is pasteurization, which utilizes high temperatures to destroy microorganisms and inactivate spoilage enzymes to prevent foodborne illness and to prolong shelf-life. While this method effectively destroys 100% of pathogenic bacteria, yeasts, and molds, high temperatures induce chemical and physical changes, altering organoleptic properties and reducing the content or extractability of some phytochemicals [3,4,5,6,7,8]. For example, pasteurization has been shown to reduce the total phenolic content, vitamin C, vitamin A, total anthocyanins, carotenoids, quercetin, kaempferol, and myricetin levels of various fruits and juices [9,10,11,12,13,14,15,16,17,18].

Therefore, there is a demand for alternative processing methods which preserve the integrity and functionality of nutrient-rich foods. High hydrostatic pressure processing (HHP) or pascalization is an established technique which utilizes high-pressure and low-heat technologies developed to preserve food products without reducing the content or extractability of their phytochemicals. In addition, this technology is more cost-efficient and environmentally friendly, and as with pasteurization, there is no need for added preservatives [8]. HHP has been found to have no impact on vitamin C content, minimal or no effect on anthocyanin content, and no effect on carotenoids (and in some cases, an increase) in some fruits and vegetables [19,20]. High pressure methods can increase the extraction of phytochemicals by accelerating mass transfer rates, which increases cell permeability and secondary metabolite diffusion [20,21].

In addition to the processing method applied, the length of time in storage can impact the nutrient density of a phytochemical-rich food [22,23,24]. There is also evidence that the type of processing method applied can impact phytochemical stability in storage. Oliveira et al. (2016) found that HHP led to better phytochemical retention during storage compared with the conventional high-heat method [4]. However, stability varies depending on the phytochemicals examined, the pressure applied, the treatment time, and the storage temperature. Understanding the stability of various phytochemicals over time and after different processing methods can improve how we evaluate the nutritional value and quality of antioxidant-rich foods in real-world conditions and help to optimize storage practices. 

While there is some overlap in the overall health-promoting effects of phytochemicals from different classes, compounds of a particular class share similar chemical structures and interact with specific molecular targets in the body, leading to different effects. Phytochemical classes that have been extensively studied for their potential health-promoting effects include terpenoids (like carotenoids), sulfuric compounds (such as isothiocyanates), and various polyphenols (e.g., anthocyanins, catechins, quercetin, and chlorogenic acid). While previously studied in isolation, there is a growing awareness that these compounds are more beneficial when consumed in their whole-food form and in complex combinations for their potential synergistic effects [25]. 

As the awareness of the health benefits linked to phytochemicals grows and nutrition guidelines emphasize diverse consumption, complex fruit and vegetable juices and smoothies have become increasingly available. However, the extractability of phytochemicals within these complex mixtures lacks sufficient evidence. Given the potential additive, synergistic, and inhibitory effects of phytochemicals in various combinations, the impacts of processing methods on complex phytochemical mixtures remains unexplored [25].

Moreover, the limited shelf-life and associated inconveniences of fresh fruits and vegetables pose a barrier to increased consumption. To address this, attempts have been made to develop shelf-stable, dry food products enriched with antioxidant-rich nutrients, offering a more convenient option for individuals seeking the health benefits of consuming fruits and vegetables. However, it is important to verify whether adequate phytochemical concentrations are present in these products, ensuring that they can effectively deliver the intended nutritional advantages they claim to offer.

This paper compares how different processing methods (pasteurization vs. HHP) affect the phytochemical concentrations of a complex mixture of fruits and vegetables and investigates how these methods influence their stability during freezing and over time in frozen storage. We also aim to test the recovery and stability of this phytochemical-rich blend after infusing it into a novel, dry-food product, called Pearls. We hypothesize that HHP will better preserve phytochemical concentrations immediately after processing, after freezing, and over time in frozen storage, and that the Pearls will provide a shelf-stable, nutrient-dense option for increasing the consumption of antioxidant-rich foods.

## 2. Materials and Methods

### 2.1. Study Design

The complex mixture created for this study contained an assortment of common yet phytochemical-rich fruits and vegetables. The ingredients were selected to represent key phytochemical classes as they are known for their abundant levels of specific compounds. Anthocyanin-rich fruits such as blueberries, blue grapes, blackberries, and raspberries were included. Carotenoids were represented by tomatoes, carrots, and red bell peppers. Isothiocyanates were incorporated through the inclusion of broccoli, cauliflower, and Brussels sprouts. Finally, apples and green tea were included to provide a source of total polyphenols, such as catechins and quercetin. Blended into a single mixture, these ingredients provided a complex blend of phytochemical compounds in which various processing and storage methods could be tested.

Viewed more holistically, apples and green tea are expected to contain catechins, quercetin, chlorogenic acid, and vitamin C; blue berries, blue grapes, blackberries, and raspberries are known to be sources of anthocyanins, quercetin, chlorogenic acid, and vitamin C; and blue grape should contain resveratrol. Tomatoes, carrots, and red bell pepper contain carotenoids, quercetin, chlorogenic acid, and vitamin C. Finally, broccoli, cauliflower, and Brussels sprouts are expected to be sources of sulforaphane, quercetin, and vitamin C, and broccoli and Brussels sprouts should contain chlorogenic acid. The specific phytochemicals tested were vitamin C, quercetin-3-glucoside, delphinidin-3-glucoside, cyanidin-3-glucoside, peonidin-3-glucoside, catechin, epigallocatechin-3-gallate, epicatechin, epicatechin gallate, chlorogenic acid, sulforaphane, resveratrol, lycopene, lutein, alpha-carotene, and beta-carotene.

For this study, phytochemical concentrations were first measured in the raw fruits and vegetables which were then blended into a mixture. The mixture was made by combining equal parts, by weight, of apples, blueberries, blue grapes, blackberries, raspberries, tomato, carrots, red bell pepper, broccoli, cauliflower, and Brussels sprouts. Two grams of dried green tea leaves were added per 100 mL water for every 100 g of a dry ingredient. This mixture was used in all the following experiments and will further be referred to as “the blend”. The blend was then poured into twelve 100 mL bottles. Four 100 mL bottles were set aside and left untreated, while four 100 mL bottles were processed by HHP and four 100 mL bottles were processed by pasteurization. One bottle from each condition was stored at 4°C and three bottles from each condition were stored at −18 °C. After freezing to −18 °C, one bottle from each condition was immediately removed from the freezer and thawed at 4 °C, which took about two days. Therefore, measurements at *t =* 0 for the fresh and frozen condition were technically made two days after processing. One month later, one bottle from each condition was removed from frozen storage to be analyzed (*t =* 1). After six months, the remaining bottles were removed and analyzed (*t =* 6). 

**Figure 1:**
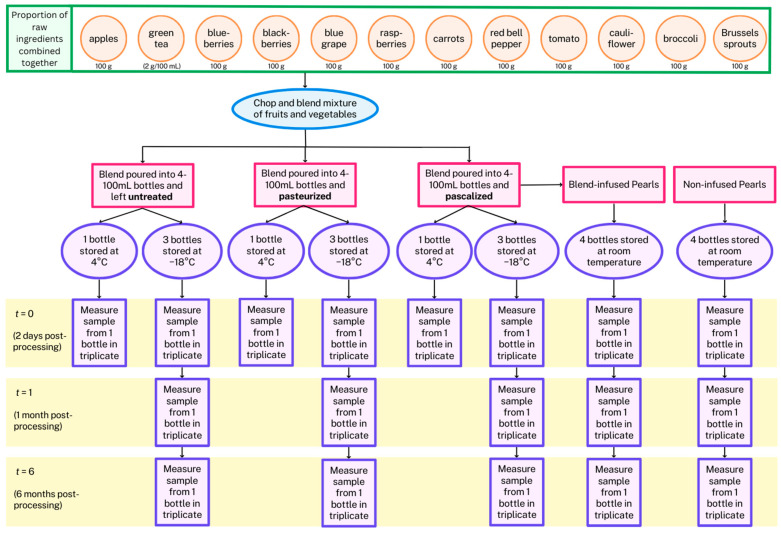
Flowchart of the experiment to test the impact of processing and storage conditions on phytochemical extractability in a complex mixture of fruits and vegetables.

A pascalized mixture was also prepared for the Pearls. This blend was infused into a novel food product made from an oat and rice mixture called a Pearl. The same phytochemicals were measured in both the non-infused Pearl and in the infused form of the Pearl to determine the phytochemical recovery in this food product, and measurements were also taken over time at one and six months to determine their stability in storage. The workflow is pictured in Figure 1.

### 2.2. Processing of the Fruit and Vegetable Blend

The raw ingredients used for this study were commercially sourced from Venlo, The Netherlands, to represent the produce accessible in the local market. The raw materials were washed at 12 °C and a vacuum was used to reduce the evaporation temperature and dry the product faster. To reduce unwanted temperature increases (>35 °C) from blending due to friction or shear forces, several components were adjusted during processing. The knives and their placement in the blender were designed to result in a rise of only 0.5 °C, to shorten processing time, and to reduce air exposure and thus oxidation. The shape of the mixing chamber and the cutter angle was also adjusted so that the mixture flows more efficiently in the vat, alternating between a 45° angle and vertical positioning during cutting. Temperature control was well-maintained below 22 °C throughout processing to prevent the degradation of phytochemical compounds. This resulted in a purple homogenous mixture where individual fruits and vegetables could not be distinguished with a drinkable consistency. Nothing was added or removed from the mixture, including water. Then, 100 mL of the blend was poured into individual plastic bottles. For pascalization, these bottles were placed in a pressurized chamber (reaching up to 6000 bar) for 3 min to kill microorganisms while keeping temperature below 25 °C. For pasteurization, the bottles were heated up to 80 °C for 3 min. Processing settings were determined based on standard practice procedures [26,27]. The Mettler Toledo FE20/EL20 pH meter (Tiel, NL) was used to measure the blend pH. A two-point calibration was performed with predefined buffer groups (4.01 and 7.00) at 25 °C and four thawed bottles of the blend were measured. 

### 2.3. Pearl Production

**Figure 2:**
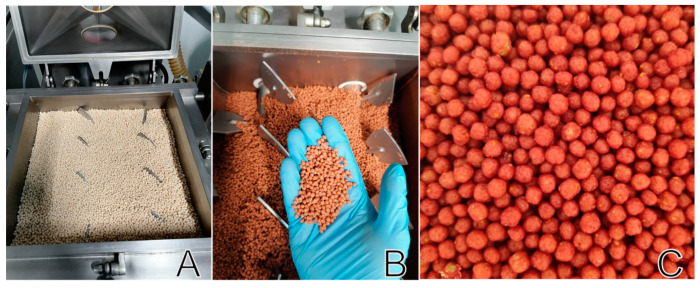
(**A**): The non-infused Pearls. (**B**): The Pearls infused with the pasteurized blend pictured with a hand for scale. (**C**): A close-up image of the infused Pearls.

The Pearls are a novel food product made from 5% oat flour and 95% rice flour mixture and formed into homogenous cereal-like spheres. Please see Figure 2 for images of the Pearls. The Pearls were charged under vacuum conditions (90 mbar) by a Dinnissen B.V. Pegasus^®^ Vacuüm Coater (Sevenum, The Netherlands) and then the pascalized blend was introduced into the kettle in a vapor form. The reduction in pressure in the kettle forced the solid particles from the blend to infuse into the grain matrix of the Pearls while the moisture evaporated. With this method, 400 g of fresh fruit and vegetables were infused into 100 g of Pearls. 

### 2.4. Phytochemical Extraction

For the extraction of polyphenols, 0.5 g of pulverized Pearls and 5 mL of water were added to centrifuge tubes and shaken manually to suspend the powder. For all samples, 10 mL of the extraction solvent (methanol (Actu-All) + 1% formic acid (Carlo Erba)) was added and mixed manually; then, the tubes were placed in an ultrasonic bath for 10 min. They were then placed on a shaker and shaken at 2500 rpm for 20 min, then centrifuged for 5 min at 4000 g. The methanol layer was then transferred to a 25 mL volumetric flask and set aside. Then, 10 mL of fresh extraction solvent was added to the centrifuge tubes, and they were placed in an ultrasonic bath for 10 min. They were then shaken again at 2500 rpm for 20 min and centrifuged for 5 min at 4000 g. The methanol layer was then transferred to the 25 mL volumetric flask and 1% formic acid in 50% methanol and 50% water was added until 25 mL was reached. The extract was then filtered through a 0.45 µm Polyethersulfone (PES) (Dispolab p/n: 210203) filter into a vial and then analyzed with HPLC using liquid-chromatography mass spectrometry (LC-MS/MS) methods. See Tables S1–S4 and Figure S1 in Supplementary Materials for system parameters, gradient liquid chromatography (LC) data, LC-MS parameters, retention times, and an example chromatogram of polyphenols via LC-MS/MS, respectively.

For the extraction of carotenoids, the same procedure applies, except that the extraction solvent used was a 60:20:20 mixture of n-heptane (Actu-All), acetone (Actu-All), and methanol (Actu-All), respectively, and the samples were centrifuged at 4600 g. The extraction solvent was added twice to increase the yield. Acetone and methanol have a stronger affinity with the water fraction and so the heptane layer remains on top. Therefore, two 10 mL additions of 60% heptane from the extraction solvent were added, with the final heptane fraction being 12 mL. A 1:1 heptane:ethanol solution with 1 g/L 5utylhydroxytoluene (CAS: 128-37-0) was added to the flask until 20 mL was reached. At this point, 13 mL was made up of a sample mixture containing water, methanol, and acetone, which was discarded. The sample was then diluted 1:1 with ethanol (Van Hees) and heptane and filtered with a 0.45 um PTFE (Polytetrafluoroethylene (PTFE), 0.45 µm, 25 mm, PP housing–Dispolab p/n: 230425) into an HPLC vial and then analyzed using liquid chromatography with ultraviolet detection (LC-UV). See Tables S5–S7 and Figures S2–S4 in Supplementary Materials for system parameters, gradient LC data, retention times, example chromatogram of lutein via LC-UV, and an example chromatogram of lycopene and beta-carotene via LC-UV, and an example chromatogram of alpha- and beta-carotene via LC-UV, respectively.

For the extraction of vitamin C, one-liter of buffer (5% meta-phosphoric acid (Van Hees), 0.05% Na2EDTA (TCI) and 5 mM Tris(2-carboxyethyl)phosphine hydrochloride (TCEP) (TCI), dissolved in milli-Q water was added to a volumetric flask. Then, 500 mg of Na2EDTA and 725 mg TCEP were dissolved in 500 mL of milli-Q water and combined with the previous solution. For the sample preparation, 2.5 g of sample and 37.5 mL of buffer were added to a 50 mL centrifuge tube and homogenized by a table vortex. This homogenate was transferred to a 50 mL volumetric flask. Then, 10 mL of additional buffer was added to the centrifuge flask and mixed manually. The extract was then filtered with 0.45 µm PES filter into a vial and then analyzed with HPLC using both LC-UV methods. See Table S8 and Figure S5 in Supplementary Materials for system parameters and an example chromatogram of vitamin C via LC-UV, respectively. 

For each extraction, the following amount of solvent was used: For the Pearls, the final volume was 25 mL (5 mL water + 2 × 10 mL extraction solvent). The same was true for the raw ingredients and the blend, with a difference of 5 g sample + 2 × 10 mL extraction liquid (filled to the 25 mL mark). For carotenoids, the volume was also 20 mL, of which 12 mL heptane was separated and filled up to 20 mL. All samples were measured in triplicate and reported in mg/kg. Raw data can be found in Table S9 in the Supplementary Materials. Error bars in all figures represent the standard deviation of triplicate sample measurements. Statistical analysis was not conducted in this experiment due to the limited sample size of one per condition, as it is generally not recommended to perform statistical analysis with only one sample per group.

## 3. Results and Discussion

### 3.1. Raw Ingredients

**Figure 3:**
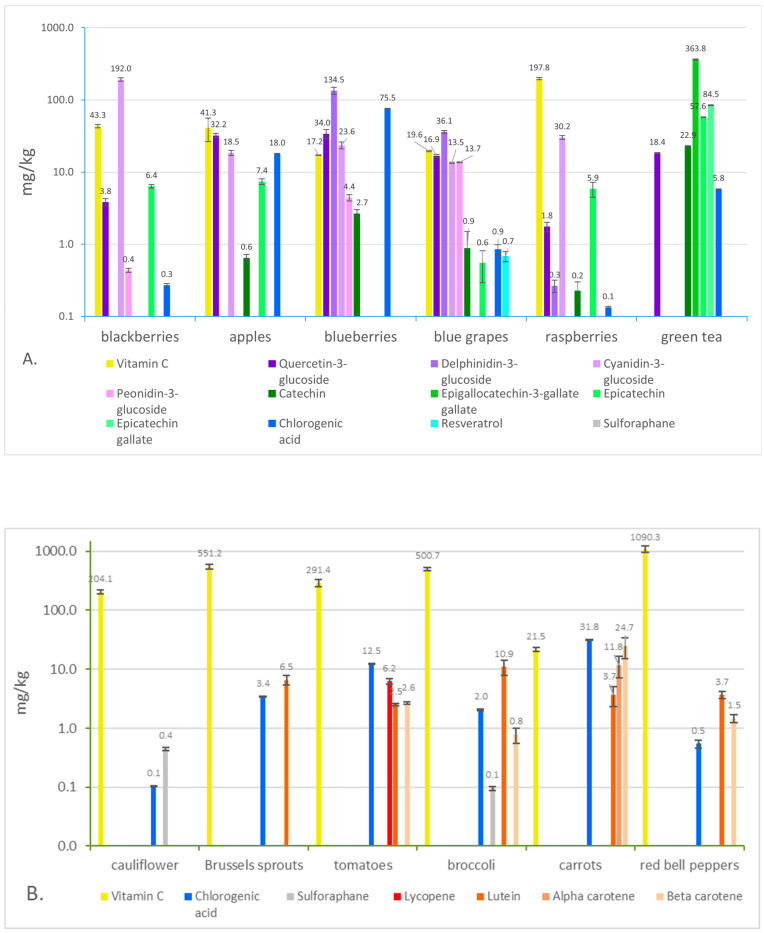
Phytochemical concentrations of raw ingredients. (**A**) Phytochemical concentrations of blackberry, apple, blueberry, blue grape, raspberry, and green tea are pictured. (**B**) Photochemical concentrations of cauliflower, Brussels sprouts, tomato, broccoli, carrots, and red bell pepper are pictured. Only vitamin C, chlorogenic acid, sulforaphane, lycopene, lutein, alpha-carotene, and beta-carotene were measured in these foods. All phytochemicals are measured in mg/kg and the *y*-axis is presented in a logarithmic scale to better display all data together.

Vitamin C and polyphenol concentrations were measured in all raw ingredients, whereas carotenoid measurements were only measured in samples where notable concentrations were expected. Phytochemical concentrations were measured in various amounts in blackberry, apple, blueberry, blue grape, raspberry, and green tea (Figure 3A). These concentrations followed expectations, with most of the anthocyanins represented in the blue and purple fruits, resveratrol most prominent in blue grape, and the catechins fully represented in green tea.

Carotenoids were measured in cauliflower, Brussels sprouts, tomatoes, broccoli, carrots, and red bell pepper (Figure 3B). In addition, prominent concentrations of carotenoids, vitamin C, and chlorogenic acid were measured, apart from the very low amount of chlorogenic acid (0.1 mg/kg) in cauliflower and sulforaphane (<0.1 mg/kg) in broccoli. Of course, plant variety, growing conditions, and the age and maturity of the plant can affect phytochemical concentrations in fruits and vegetables, leading to variations. 

### 3.2. Blend-Infused Pearl Stability:

**Figure 4:**
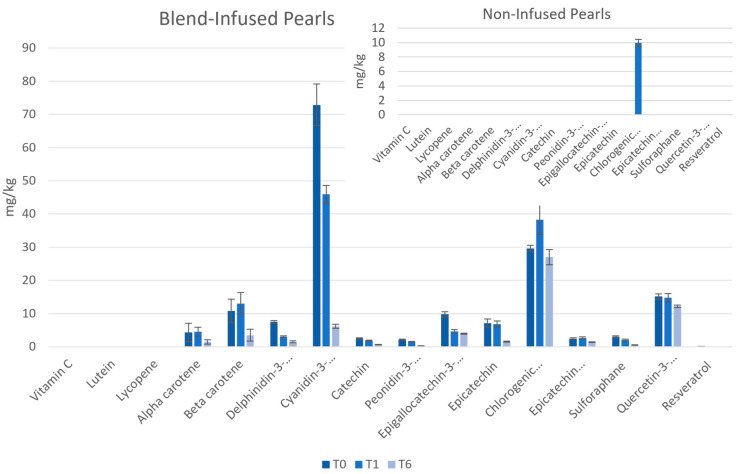
Bioactive content and stability in blend-infused and non-infused Pearls. All phytochemicals were measured in mg/kg. T0 = time 0, T1 = one month, and T6 = six months. Vitamin C was measured in mg/kg. The only phytochemical detected in the non-infused pearls was chlorogenic acid.

Phytochemical concentrations were measured in non-infused Pearls and blend-infused Pearls over time to test their stability at room temperature, as seen in Figure 4. The non-infused Pearls, which consisted of only a mixture of oats and rice, only showed notable concentrations of chlorogenic acid. This can be accounted for by the chlorogenic acid present in grains such as oats and rice. The amount of chlorogenic acid in the blend-infused Pearls was about three times greater than the non-infused, likely due to contributions from the infused blend. Lutein, lycopene, and vitamin C were not measured at detectable levels in the blend-infused Pearls, which could be the result of losses during the vaporization or infusion step, although temperatures did not exceed 35 °C. The results over time suggest that while shelf-stable after one month in most phytochemicals aside from delphinidin-3-glucoside, cyanidin-3-glucoside, and epigallocatechin-3-gallate, the extractable fraction of phytochemicals in the blend-infused Pearls was diminished for most compounds at six months.

**Figure 5:**
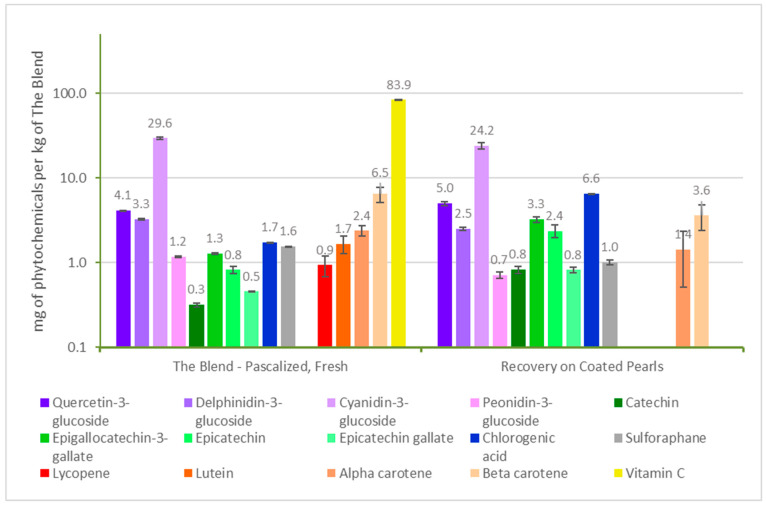
The blend in whole food and dry food formats. The phytochemical content of the fresh, pascalized fruit and vegetable blend are compared with the phytochemical content found in the same amount by weight of pascalized fruit and vegetable coating added to the Pearls. The chlorogenic acid levels contained in the oat–rice mixture portion of the Pearls were controlled for. All phytochemicals are measured in mg/kg and data are presented on a logarithmic scale.

In Figure 5, the blend-infused Pearls are compared with the fresh, pasteurized blend, which was the same formulation that was infused into the Pearls. For the same amount by weight of fruits and vegetables, the Pearl coating measured only a subtle loss in the recovery of anthocyanins, sulforaphane, and carotenoids. Interestingly, the additional processing step to vaporize and infuse the blend led to an increased extraction of quercetin-3-glucoside, catechin, epicatechin-3-gallate, epicatechin, and chlorogenic acid. Overall, 33 g of condensed coating was produced for every 400 g of fruits and vegetables, which was then applied to 100 g of an oat–rice food product. This resulted in a food product weighing 133 g containing comparable amounts of phytochemicals as 400 g of whole fruit and vegetables. Although there was a loss in sensitive compounds such as vitamin C, lutein, and lycopene, the Pearl production method was effective at concentrating the phytochemicals from the whole food mixture onto a dry product.

### 3.3. Impact of Processing

#### 3.3.1. Vitamin C

Fresh, untreated, and pascalized samples did not differ in vitamin C content after processing, whereas pasteurized samples decreased by 39%. For samples which were immediately frozen and defrosted after processing, vitamin C concentrations in pascalized and pasteurized samples were 9% and 53% lower than untreated samples, respectively. Freezing and thawing the samples immediately after processing also altered the vitamin C content of all the samples compared with those just kept at 4 °C. Freezing and thawing immediately increased the vitamin C content in all conditions (untreated samples increased by 70%, pascalized by 55%, and pasteurized by 29%).

**Figure 6:**
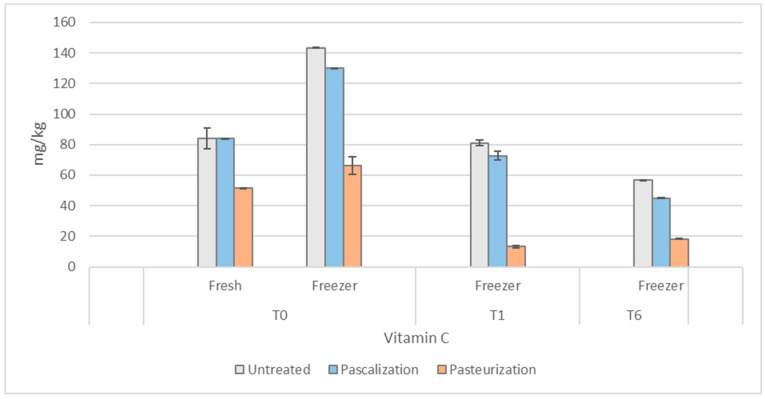
Vitamin C concentrations in the blend after various processing methods. T0 = time 0, T1 = one month, and T6 = six months. Vitamin C was measured in mg/kg.

After one month in the freezer, the untreated and pascalized samples subsequently decreased by 44% and 43%, respectively, but to a level similar to that of the fresh untreated or pascalized samples immediately after processing. The frozen pasteurized samples decreased in vitamin C after one month by 80%, measuring 84% lower compared with the untreated samples at one month. All the samples continued to decrease up to six months. The untreated, pascalized, and pasteurized samples decreased by 61%, 65%, and 72%, respectively, at six months. The results for vitamin C can be seen in Figure 6.

Vitamin C is highly water-soluble, and its degradation is sensitive to temperature, light, pH, oxygen, and trace metal ion concentration. Its high sensitivity to processing lends it as a suitable surrogate marker for phytochemical degradation in a food product. However, the presence of certain phenols (e.g., flavonols such as quercetin) or sugars, which occur in these complex mixtures, decrease oxygen solubility, and therefore protect vitamin C from being oxidized [22]. In keeping with our results, previous studies have also found that frozen-stored samples had higher levels of vitamin C than fresh, possibly attributable to arrested enzymatic activity and slowed oxidation over time [23]. The immediate increase in vitamin C concentration after freeze-thawing may be related to the breakdown of cell walls during the freezing process, making it more available and easier to extract. However, continued freeze–thaw cycles (which were avoided in this study), have been shown to result in a significant loss of vitamin C [24]. 

#### 3.3.2. Carotenoids (Lutein, Lycopene, Alpha-Carotene, Beta-Carotene)

**Figure 7:**
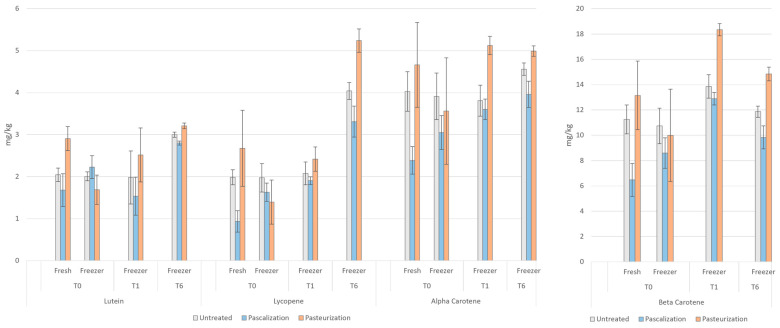
Carotenoid concentrations in the blend after various processing methods. T0 = time 0, T1 = one month, and T6 = six months. Lutein, lycopene, alpha-carotene, and beta-carotene were measured in mg/kg.

The immediate freezing and thawing of pasteurized samples decreased their concentration by 24–48%, whereas the pascalized samples increased 28–74% for all carotenoid measurements. The untreated samples decreased mildly, less than 5% for all carotenoids after immediate freeze–thawing. For nearly all time points, the pascalized samples contained lower carotenoid concentrations than the untreated samples, whereas the pasteurized samples were higher in carotenoid concentrations than the untreated samples. After one and six months at −18 °C, the pascalized samples increased in carotenoid concentration by 17–50% and 14–103%, respectively, compared with *t =* 0 (except for lutein at *t =* 1). The pasteurized samples also increased at one month by 44–84% and at six months by 40–276%. The untreated samples did not differ much at *t* = 1 compared with *t =* 0, aside from beta-carotene, which increased 29%. The untreated samples increased similarly to that of the pascalized samples at six months, by 11–105%. The results for carotenoids can be seen in Figure 7.

The higher carotenoid content that we found in our pasteurized samples is consistent with a study by Leong and Oey (2012) who tested the effect of processing on carotenoids in summer fruits and vegetables [28]. They reported that heating increased the chemical extractability of phytochemical compounds due to the release of phytochemicals from chromoplasts, leading to an increase in their concentration. High heat cooking methods such as boiling and grilling were also found to increase lutein and lycopene content compared with lower heat methods such as steaming and microwaving. Lemmens et al. (2011) found that beta-carotene bioaccessibility in carrots increased with increasing intensity of mechanical processing and increasing intensity of thermal processing [29]. 

Adkison et al. (2018) reported that apricots exhibited large increases in beta-carotene after freezing (35%) [30]. Like our results, they reported a continued rise in beta-carotene (56%) after three months in frozen storage compared with fresh. However, in their study, frozen apricots showed an increase in beta-carotene content compared with fresh samples and this increased during the storage period [31]. 

Differences in carotenoid extractability can depend on the intactness of the cellular matrix in fruits and vegetables and the rate at which carotenoid–protein complexes, such as those in green leafy vegetables, or those in semi-crystalline forms, such as those found in roots and fruits such as carrots and tomato, are disrupted under high heat conditions such as processing or cooking [32,33]. The higher stability of carotenoids over time can also be related to their ability to form these semi-crystalline structures bound to plastid-derived membranes or sub-cellular lipid structures and proteins [34,35]. Food processing which modifies the matrix structure by mechanical homogenization or heat treatment has been reported to have a beneficial impact on the extractability of carotenoids from different foodstuffs, which may depend on their lipophilicity [33,36]. For example, the extractability of more hydrophilic carotenoids, such as lutein, seems higher and less dependent on plant cell matrix disruption [37]. Results are, however, not always consistent and identifying the optimal conditions for carotenoid preservation or maximation in a complex mixture is difficult based on the existing data [33,36].

### 3.4. Phenolic Compounds/Total Polyphenols

**Figure 8:**
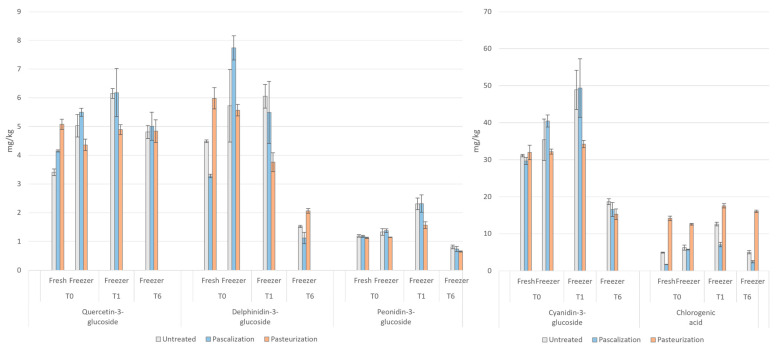
Polyphenol concentrations in the blend after various processing methods. T0 = time 0, T1 = one month, and T6 = six months. Cyanidin-3-glucoside, quercetin-3-glucoside, delphinidin-3-glucoside, peonidin-3-glucoside, and chlorogenic acid were measured in mg/kg.

The oxidation and loss of phenolic compounds can occur at different stages of food processing. Since phenolic compounds are heavily concentrated in fruit and vegetable peels, stems, and seeds, a great deal are lost if peeling or seed removal steps are included in processing [24]. Blanching, which often occurs before canning and freezing, causes phenolic compound loss via water solubility and can inactivate enzymes leading to phenolic oxidation [38]. During storage, chemical degradation can occur due to oxygen and light exposure. In this study, peeling, seed-removal, and blanching did not occur, so the phenolic gains or losses can be largely explained by the processing method implemented and the length of time in storage. The results for phenolic compounds can be seen in Figure 8.

*Anthocyanins (Cyaninidin-3-glucoside, delphinidin-3-glucoside, peonidin-3-glucoside)*—Anthocyanins are abundant flavonoids which give fruits and vegetables their red, purple, blue, and black hues. Their intake has been shown to improve carbohydrate metabolism and decrease the risk of developing metabolic disorders [15,39]. In our study, anthocyanin content increased to a notable degree after immediate freezing and thawing in the pascalized samples (by 37% for cyanindin-3-glucoside, 136% for delphinidin-3-glucoside, and 17% for peonidin-3-glucoside), but had little to no effect on untreated and pasteurized samples, respectively. At one month, both the pascalized and pasteurized samples were higher in anthocyanin content than the untreated samples and to a similar degree (by 44–48%). Most samples, whether pascalized or pasteurized, increased in anthocyanin content at one month (by 9–57%), compared with the untreated samples whose content decreased at one month from *t =* 0 (by 39–73%). At six months, nearly all the samples of each processing method had decreased below their *t =* 0 values to similar degrees. 

Some studies have shown significant anthocyanin degradation in mulberries, blackcurrant juice, and blueberry juice from high heat exposure [15,40,41]. Alternatively, the increase seen in our pascalized samples and over time has been previously reported and was likely related to the inactivation from the high pressures of enzymes such as β-glucosidase, peroxidase, and polyphenol oxidase which release anthocyanins from sugar molecules or prevent their oxidation [42,43]. The increases in anthocyanin content seen at one month for the pasteurized and pascalized samples (in all but delphinidin-3-glucoside in pascalized samples) may be related to the degradation of the plants’ cellular structures at −18 °C, resulting in a higher extractability of anthocyanins and other polyphenols. The decrease in anthocyanin content after one month may be due to the hydroxyl group on the phenolic substances becoming susceptible to oxidation [41].

*Flavonol (Quercetin)*—Quercetin is a flavonoid present in many fruits, vegetables, and grains and has been studied for its potential health benefits regarding antioxidant capacity, anti-inflammatory activity, cardiovascular health, brain health, and the immune system [44,45,46,47]. There are inconsistencies in the literature regarding the effect of pasteurization and pascalization on quercetin levels [31,48,49]. In this study, quercetin-3-glucoside increased by 22% in the pascalized samples and by 49% in the pasteurized samples compared with the untreated. Immediate freezing and thawing resulted in the pascalized samples showing 9% more quercetin-3-glucoside than the untreated samples, and the pasteurized samples showing 13% less. After one month at −18 °C, quercetin-3-glucoside was 26% higher in both the pascalized and pasteurized samples compared with the untreated, but at six months, levels in the processed samples were comparable with the untreated at six months. Looking at the processing method over time, the pascalized samples increased in quercetin-3-glucoside content after one month of storage at 18 °C, but levels decreased by 9% from *t* = 0 at six months. Although measuring the lowest among the frozen samples at *t =* 0, the pasteurized samples increased in quercetin-3-glucoside levels by 41% at one month and remained 11% higher than its *t =* 0 at six months in 18 °C storage. Overall, pascalization was the optimal method for preserving quercetin-3-glucoside levels in frozen storage, from *t =* 0 to *t =* 6.

*Phenolic acids (Chlorogenic acid)*—Chlorogenic acid is one of the most abundant, highly functional polyphenolic compounds in the human diet, and its intake is inversely correlated with metabolic syndrome and chronic disease risk [50]. In a study by Oliveira et al. on the effect of frozen storage and pasteurization on peach phytochemical content, they found that the extractability of chlorogenic acid increased (38% and 24%) after the high heat processing of fresh samples and samples frozen for 360 days [4]. The same has been seen in high temperature processing studies on green coffee beans and artichokes [41,51,52]. In our experiment, we saw a marked 188% and 100% increase in chlorogenic acid after pasteurization of the fresh and frozen samples and, even at six months, chlorogenic acid in the pasteurized samples was measured 220% higher than in the untreated samples. Despite these consistencies, findings are not consistent in the literature and seem to vary depending on the specific food matrixes and processing conditions involved.

*Stillbenes (Resveratrol)*—Resveratrol is sensitive to light, pH, and increased temperature because of its unstable hydroxyls and carbon double bond. Scientists have experimented with co-encapsulation with ultrasound treatment, protective supermolecules, and inclusion complexes to improve its stability, but there are very limited studies focusing on the optimal processing methods for resveratrol. In our processing experiment, resveratrol was measured for each sample, but was not detected above 0.4 mg/kg, so no conclusions can be made regarding this phytochemical. 

### 3.5. Catechins

**Figure 9:**
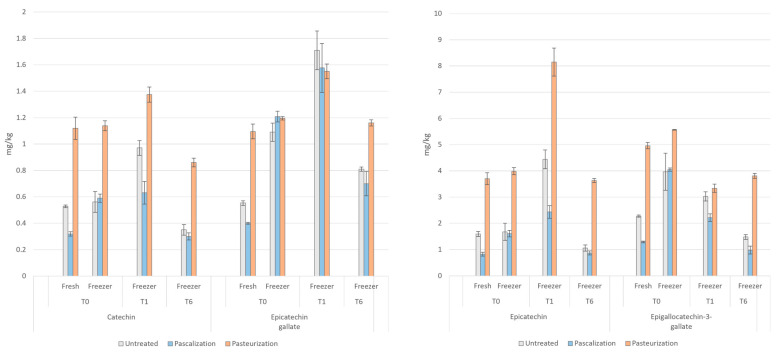
Catechin concentrations in the blend after various processing methods. T0 = time 0, T1 = one month, and T6 = six months. Catechin, epicatechin, epicatechin gallate, and epigallocatechin-3-gallate were measured in mg/kg.

The levels of various catechins in the samples after each processing method and storage time generally followed a similar pattern. As seen in Figure 9, samples that were pasteurized measured highest in various catechins (as much as 157% higher than untreated), except for epicatechin gallate at *t =* 0 and *t =* 1 from frozen samples where pascalized samples had the highest content. Immediate freezing and thawing increased various catechin content in all the processed samples. At six months, for samples that underwent the same processing method, all catechins decreased in all samples below that of their frozen *t =* 0 values.

Catechins are a type of flavonoid called flavanols which are abundant in tea leaves, especially green tea, and to a smaller degree in cocoa, berries, and apples. They have been studied for their health benefits, including antioxidant and anti-inflammatory effects, as well as their potential benefits for heart health, brain function, and cancer prevention [53]. Catechins are sensitive to oxidation by heat, enzymatic activity, and basic environments [54]. In the presence of the enzyme PPO, catechins are oxidized to form theaflavins and thearubigins. Controlling the pH and temperature can minimize oxidation, as PPO activity is optimized at pH 5.5 and 40 °C in fresh tea leaves [55].

Since catechins are very unstable in alkaline solutions (pH > 8), acids are often added to improve their stability [55]. The pH of the blend was measured at 3.86 ± 0.05, so our mixture of fruits contributed to the lower pH environment and likely increased the stability of the catechins. Other studies have also seen an increase in the concentration and extractability of catechins following pasteurization [56]. According to previous research, the dominant reactions affecting catechin concentrations after pasteurization are thermal degradation and the depolymerization of higher oligomeric and polymeric procyanidins into dimers and trimers which increase procyanidin content [56]. 

### 3.6. Sulforaphane

**Figure 10:**
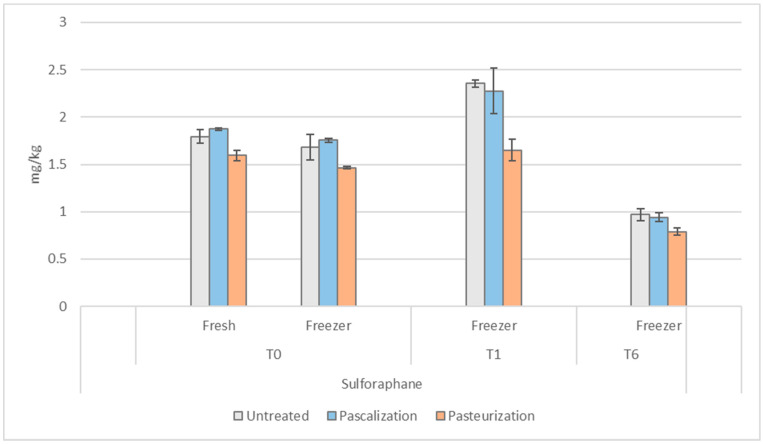
Sulforaphane concentrations in the blend after various processing methods. T0 = time 0, T1 = one month, and T6 = six months. Sulforaphane was measured in mg/kg.

The effect of immediate freezing and thawing on sulforaphane levels was consistent despite the processing method (−6% for pascalized and untreated samples, and −8% for pasteurized) at *t =* 0, as seen in Figure 10. The pasteurized samples at *t =* 0 were 11% lower in sulforaphane than the untreated in fresh samples and 13% lower in frozen. At one month in the freezer, the levels of sulforaphane increased in each processing method from *t =* 0: in the untreated by 18%; in the pascalized by 57%; and in the pasteurized by 94%. At six months, sulforaphane levels in all the samples decreased below their *t =* 0 levels: in the untreated by 31%; in the pascalized by 35%; and in the pasteurized by 35%. 

Glucosinolates are sulfur-containing compounds found in cruciferous vegetables that can be hydrolyzed by myrosinases to form reactive metabolites such as isothiocyanates. Myrosinases are separated from glucosinolates in intact plant cells and do not come in contact unless cells are damaged. They are also inactivated at 60 °C, preventing the conversion of glucosinolates to their bioactive metabolites [57]. Glucoraphanin is a glucosinolate that is hydrolyzed to the isothiocyanate sulforaphane, which has been studied for its antioxidant, anti-inflammatory, and anticancer activity [1]. While glucosinolates are present in high concentrations in cruciferous vegetables, processing and preparation methods affect the amount of isothiocyanates that are formed from glucosinolate-rich foods.

We expect the chopping steps to create the fruit and vegetable blends to sufficiently expose the glucosinolates in the broccoli, cauliflower, and Brussels sprouts to myrosinases, forming the bioactive metabolites. Subsequently, the only samples exposed to heat above 35 °C were the pasteurized samples, which could have inactivated myrosinases and prevented further conversion during the mixing steps used during measurement. This could explain the lower sulforaphane concentrations measured in pasteurized samples. 

At one month, there was a rise in sulforaphane for all treatment types. Other studies have also seen the highest sulforaphane and glucoraphanin levels after 20 days in storage, although their pasteurized samples were two-fold reduced [58]. It has also been reported that fresh-cut broccoli releases ethylene, which can trigger glucoraphanin biosynthesis and subsequent sulforaphane formation [59]. 

## 4. Conclusions

Overall, the results from this study have shown that the applied processing and storage method affects the extractability and stability of phytochemicals in a complex mixture of fruits and vegetables, which is likely to impact their potential health-promoting properties. Contrary to our initial hypothesis regarding the ability of HHP technologies to better preserve the concentration of phytochemical concentrations in a complex fruit and vegetable mixture, the optimal processing method seems to vary based on the phytochemical of interest. 

Pascalization better preserved vitamin C (−0.3%), quercetin-3-glucoside (+22%), and sulforaphane (+4.3%) compared with the untreated samples, whereas pasteurization resulted in higher concentrations of chlorogenic acid (+188%), carotenoids (+16–42%), and catechins (+98–132%) compared with the untreated samples of the fresh blend. The immediate freeze–thawing of the pascalized samples was also the optimal treatment for higher contents of lutein (+33%), lycopene (+74%), cyanidin-3-glucoside (+37%), delphinidin-3-glucoside (+136%), peonidin-3-glucoside (+17%), and epicatechin gallate (+202%). 

After one and six months in frozen storage (−18 °C), the pascalized samples had the highest concentrations of vitamin C (T1 = +450%, T6 = +146%), quercetin-3-glucoside (T1 = +0.5%, T6 = +3%), peonidin-3-glucoside (T1 = +0.03%, T6 = +13%), cyanidin-3-glucoside (T1 = +1%, t6 = +8%), and sulforaphane (T1 = +0.5%, t6 = +3%) compared with pasteurization. Alternatively, the pasteurized samples measured highest in carotenoids (T1 = +27–64%, T6 = +15–58%), chlorogenic acid (T1 = +44%, t6 = +84%), and catechins (T1 = +8–45%, T6 = +40–76%) at one month and six months compared with the pascalized samples. The pascalized samples, after one month at −18 °C, had the highest delphinidin-3-glucoside content (+46% compared with pasteurized), whereas the pasteurized samples measured higher levels by month six (+46% compared with pascalized). 

Additionally, the infusion of the blend onto the Pearls revealed to be an effective method of transferring many phytochemical compounds from a fruit and vegetable mixture onto a dry food product. The stability test, however, showed that most compounds notably degraded by six months, and storage methods to prevent oxidative breakdown, for example, are worth investigating. The variety of fruit and vegetable and cultivar can also impact phytochemical concentration and should also be considered when aiming to optimize the health promoting food products derived from fruits and vegetables. Given the complex responses of processing methods on various phytochemical compounds, these impacts should be considered to produce foods aimed at aiding the prevention of chronic disease development.

## Data Availability

All of the data is contained within the article and the supplementary materials.

## Supplementary Materials

The following supporting information can be downloaded at: https://www.mdpi.com/article/10.3390/antiox12061252/s1, Table S1: System parameters; Table S2: Gradient LC; Table S3: LCMS parameters; Table S4: Compound table with indicative retention times; Table S5: System parameters; Table S6: Gradient LC; Table S7: Compound table with indicative retention times; Table S8: System parameters; Table S9: Raw Data Overview. Figure S1: Example chromatogram of standard LC-MS/MS; Figure S2: Example chromatogram of standard lutein UV (450 nm); Figure S3: Example chromatogram of standard lycopene and beta-carotene UV (450 nm); Figure S4: Example chromatogram of sample UV (450 nm); Figure S5: Example chromatogram of sample UV (254 nm).
