# Supplementary material for: Impact of Processing Method and Storage Time on Phytochemical Concentrations in an Antioxidant-Rich Food Mixture
Source: Antioxidants (Basel). 2023 Jun 10;12(6):1252. doi: 10.3390/antiox12061252 (PMC10295423; doi:10.3390/antiox12061252)
Supplement: Supplementary file 1 [file antioxidants-12-01252-s001.zip › Supplementary Files_Figures S1-S5.pdf]

Supplementary Materials –Figures:

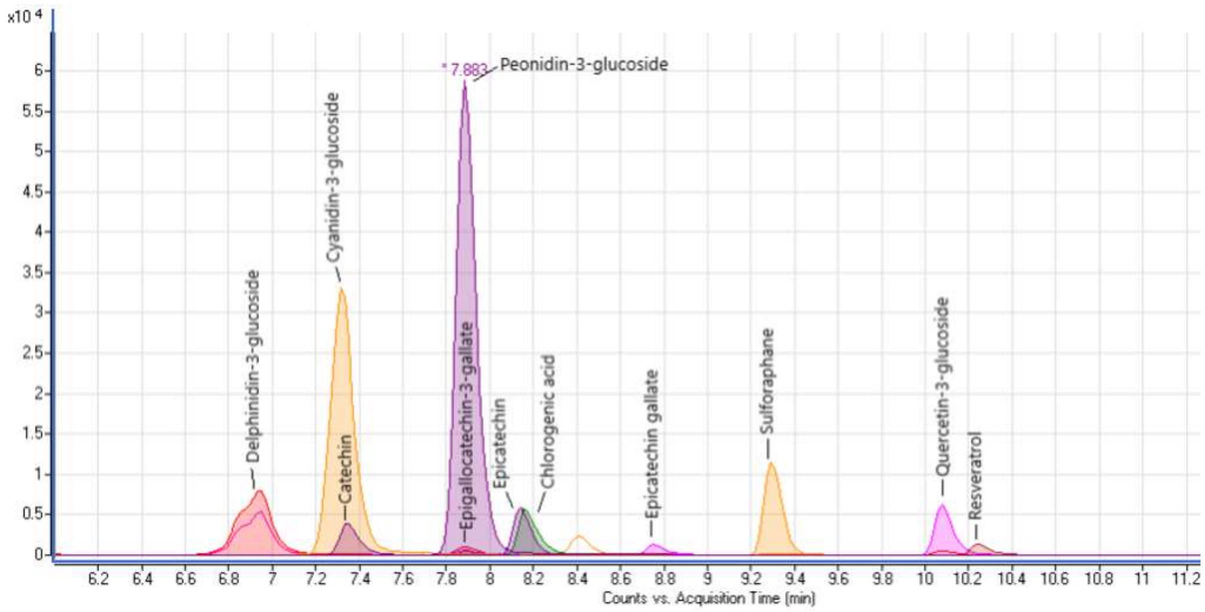

Figure S1: Example chromatogram of standard LC-MS/MS

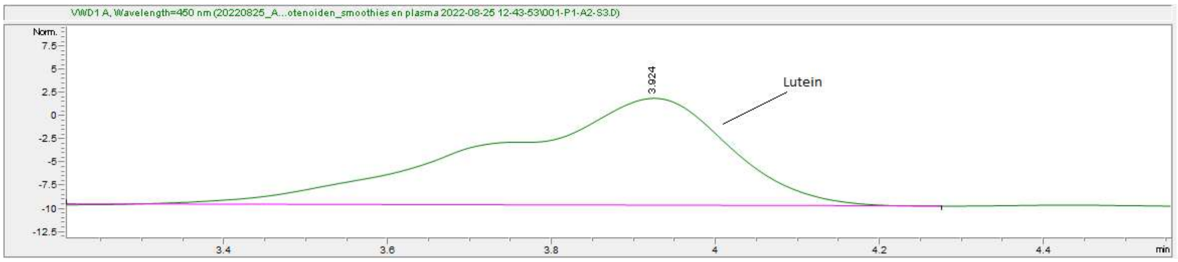

Figure S2: Example chromatogram of standard lutein UV (450 nm)

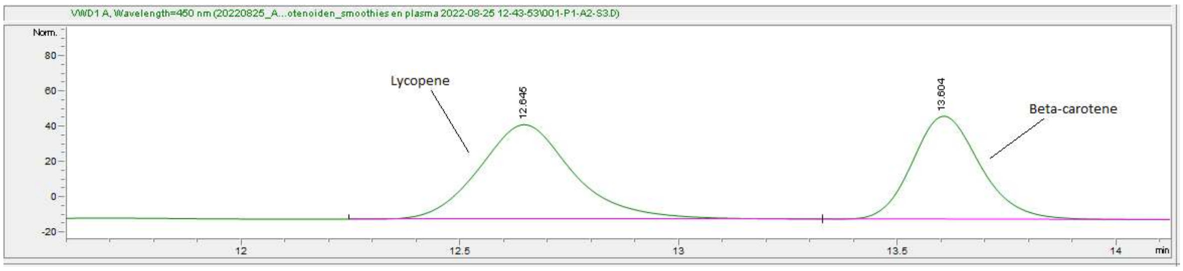

Figure S3: Example chromatogram of standard lycopene and beta-carotene UV (450 nm)

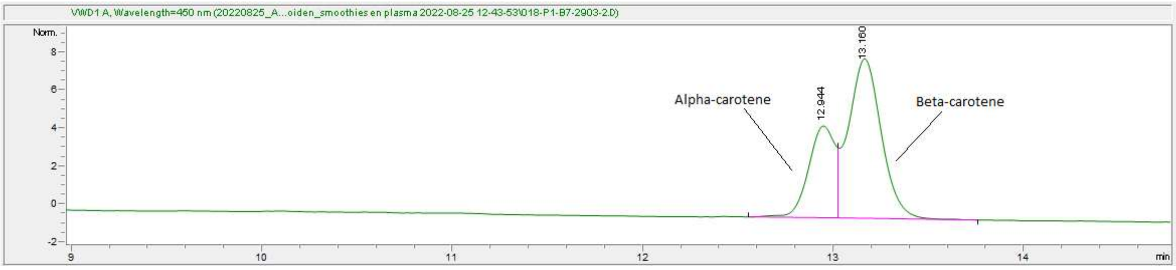

Figure S4: Example chromatogram of sample UV (450 nm)

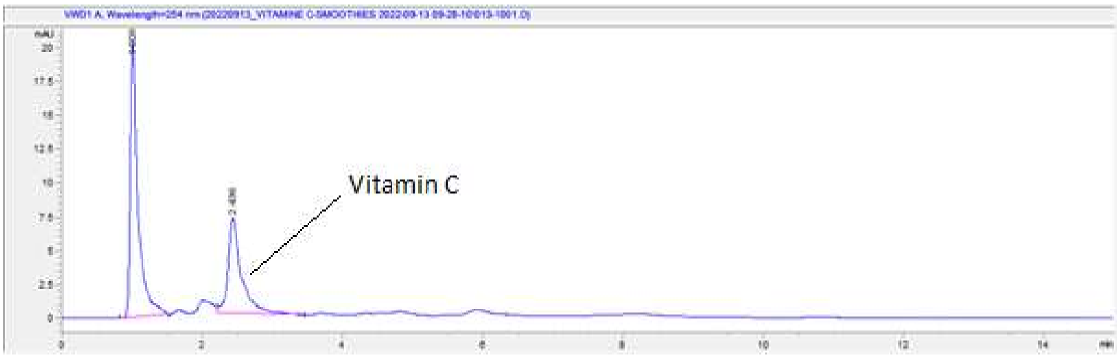

Figure S5: Example chromatogram of sample UV (254 nm)
