# Supplementary material for: Impact of Processing Method and Storage Time on Phytochemical Concentrations in an Antioxidant-Rich Food Mixture
Source: Antioxidants (Basel). 2023 Jun 10;12(6):1252. doi: 10.3390/antiox12061252 (PMC10295423; doi:10.3390/antiox12061252)
Supplement: Supplementary file 1 [file antioxidants-12-01252-s001.zip › Supplementary Materials_Tables S1-S9.pdf]

Supplementary Materials –Tables:

LC-MS/MS method for polyphenols:

Table S1: System parameters

Instrument LC:	LC-MS/MS
Column:	C18 2,5µm (4,6 mm x 150 mm)
Flow:	0.75 mL/min
Stop time:	20 min
Post time:	0 min
Injection volume:	10 µL
Column temperature:	45 °C
Sampler temperature:	15 °C
UV Detector:	280 nm
Ionization mode:	ESI
Gas temperature:	300 °C
Gas flow:	10 L/min
Sheath gas temperature:	350 °C
Sheath gas flow:	11 L/min
Capillary voltage:	3500 V
VCharging:	500
Detection mode:	MRM
Mobile phase A:	1% formic acid in ULC-MS water
Mobile phase B:	1% formic acid in ULC-MS methanol

Table S2: Gradient LC

**Time (min)**		**Mobile phase (v/v %)**	
		**A**	**B**
0		95	5
2.0		95	5
13.0		0	100
15.0		0	100
16.0		95	5
20.0		95	5

Table S3: LCMS parameters

Compound	Precursor ion	Product ion	Dwell time (ms)	Frag (V)	CE (V)	CA (V)	Polarity
Delphinidin-3-glucoside	465.4	303	50	149	24	4	Positive
Delphinidin-3-glucoside	465.4	229	50	149	50	4	Positive
Cyanidin-3-glucoside	449.4	287.1	50	148	20	4	Positive
Cyanidin-3-glucoside	449.4	137.1	50	148	50	4	Positive
Catechin/Epicatechin	291.3	139	50	97	12	4	Positive
Catechin/Epicatechin	291.3	123	50	97	32	4	Positive
Peonidin-3-glucoside	463.4	301.1	50	128	20	4	Positive
Peonidin-3-glucoside	463.4	286	50	128	48	4	Positive
Epigallocatechin-3-glucoside	459.4	151.2	50	118	8	4	Positive
Epigallocatechin-3-glucoside	459.4	139.1	50	118	24	4	Positive
Chlorogenic acid	355.3	163.1	50	82	12	4	Positive
Chlorogenic acid	355.3	135	50	82	40	4	Positive
Epicatechin gallate	443.4	139.2	50	108	28	4	Positive
Epicatechin gallate	443.4	123	50	108	24	4	Positive
Sulforaphane	178.3	114.1	50	97	8	4	Positive
Sulforaphane	178.3	72	50	97	32	4	Positive
Quercetin-3-glucoside	465.4	303.1	50	103	8	4	Positive
Quercetin-3-glucoside	465.4	85	50	103	28	4	Positive
Resveratrol	229.2	135	50	103	12	4	Positive

Table S4: Compound table with indicative retention times

Compound	Retention time – indicative (min)
Delphinidin-3-glucoside	7.0
Cyanidin-3-glucoside	7.4
Catechin	7.4
Peonidin-3-glucoside	7.9
Epigallocatechin-3-gallate	7.9
Epicatechin	8.2
Chlorogenic acid	8.2
Epicatechin gallate	8.8
Sulforaphane	9.3
Quercetin-3-glucoside	10.1

Resveratrol	10.3

LC-UV method for carotenoids:

Table S5: System parameters

Instrument LC:	LC-UV
Column:	C18 2.5 µm (3.0 mm x 150 mm)
Flow:	0.5 ml/min
Stop time:	28 min
Post time:	0 min
Injection volume:	5µL
Temperature sampler:	15 °C
Temperature oven:	40 °C
UV detector:	450 nm
Mobile phase A:	97.5% methanol, 2.5% ULC-MS water
Mobile phase B:	100% Isopropyl alcohol

Table S6: Gradient LC

**Time (min)**	**Mobile phase (v/v %)**	
	**A**	**B**
0	100	0
3.0	100	0
14.0	25	75
17.0	0	100
20.0	0	100
22.0	100	0
28.0	100	0

Table S7: Compound table with indicative retention times

**Compound**	**Retention time – indicative (min)**
Lutein	3.4
Lycopene	12.6
Alpha-carotene	13.5
Beta-carotene	13.6

LC-UV method for vitamin C:

Table S8: System parameters

Instrument LC:	LC-UV
Column:	C_{18} T3 3µm, 4.6 x 150 mm
Flow:	0.5 ml/min
Stop time:	15 min
Post time:	0 min
Injection volume:	5µL
Temperature sampler:	15 °C
Temperature oven:	20 °C
UV detector:	254 nm
Gradient:	Isocratic
Mobile phase	0.05% formic acid in ULC-MS water

Table S9: Raw Data Overview

A1. Polyphenol Measurements

t0		c. (mg/kg)										
Naam	BL-nummer	**Delphinidin-3-glucoside**	**Cyanidin-3-glucoside**	**Catechin**	**Peonidin-3-glucoside**	**Epigallocatechin-3-gallate**	**Epicatechin**	**Chlorogenic acid**	**Epicatechin gallate**	**Sulforaphane**	**Quercetin-3-glucoside**	**Resveratrol**
Pas-k	2950-1	3.326112	28.59499	0.337752	1.158937	1.324091	0.910097	1.788841	0.451935	1.555873	4.108685	0.046687
Pas-k	2950-2	3.296751	29.90546	0.304295	1.144568	1.258205	0.780353	1.681147	0.464693	1.539565	4.15798	0.032541
Pas-k	2950-3	3.212982	30.44242	0.31563	1.227824	1.290723	0.782	1.723959	0.462778	1.562252	4.181563	0.041918
Pst-k	2938-1	5.774619	31.33584	1.111834	1.097067	4.823085	3.520709	13.52332	1.032095	1.284252	4.927313	0.026725
Pst-k	2938-2	5.765444	30.45652	1.03901	1.118639	5.002411	3.626918	14.26237	1.119693	1.307593	5.037793	0.026486
Pst-k	2938-3	6.413337	34.20742	1.207328	1.154587	5.056879	3.95822	14.68234	1.13451	1.369659	5.268477	0.027611
o-k	2942-1	4.535947	30.75845	0.527371	1.251873	2.319057	1.667127	5.067288	0.565963	1.514324	3.37845	0.059363
o-k	2942-2	4.436938	31.44049	0.538857	1.171275	2.27238	1.486087	4.873496	0.533374	1.41729	3.307029	0.018579
o-k	2942-3	4.49108	31.20294	0.518183	1.14252	2.234021	1.629351	4.806855	0.560568	1.532221	3.536794	0.02404
Pas-v 1	2951-1	8.136392	40.42552	0.591716	1.279988	4.028006	1.466904	5.665171	1.165999	1.431855	5.513425	0.016809
Pas-v 1	2951-2	7.29325	38.85501	0.557892	1.439365	4.121074	1.705018	5.73285	1.212252	1.466007	5.350382	0.02554
Pas-v 1	2951-3	7.790111	42.142	0.619673	1.399441	4.003325	1.646758	5.850506	1.247342	1.464756	5.629246	0.040828
Pst-v 1	2939-1	5.494203	32.27339	1.177736	1.141303	5.582181	4.12913	12.64487	1.209776	1.214823	4.592741	0.037696
Pst-v 1	2939-2	5.414909	31.46356	1.102753	1.153961	5.555918	3.973406	12.74626	1.191902	1.201838	4.223755	0.03853
Pst-v 1	2939-3	5.795974	32.86476	1.133587	1.14406	5.555394	3.859585	12.31941	1.184226	1.228432	4.266781	0.033768
o-v 1	2943-1	4.268446	29.01951	0.476157	1.162079	3.173914	1.328617	5.526311	1.044055	1.268287	4.583451	0.014981
o-v 1	2943-2	6.341315	38.08226	0.578718	1.378189	4.187413	1.715498	6.415796	1.055756	1.447958	5.192672	0.016937

o-v 1	2943-3	6.55719	39.13988	0.629004	1.445712	4.537789	1.9772	6.899347	1.169575	1.466418	5.305689	0.021497
bramen	2899-1	0.120137	182.4787	0.144575	0.419998	0.08049	6.000777	0.341085	0.019542	0.000252	3.624236	0.006108
bramen	2899-2	0.064182	203.2943	0.168175	0.474497	0.031817	6.69808	0.244498	0.020302	0.00011	4.3418	0.011835
bramen	2899-3	0.032173	190.1798	0.160253	0.411721	0.024269	6.431548	0.226417	0.012803	0.000149	3.543817	0.007399
appel	2895-1	0.016935	20.09198	0.580097	0.025986	0.012698	6.623006	15.93164	#VALUE!	0	31.18786	0.015076
appel	2895-2	0.011661	17.0368	0.638226	0.032245	0.012641	7.969521	19.27321	#VALUE!	0	34.65237	0.019935
appel	2895-3	0.010472	18.52009	0.724295	0.029202	0.005176	7.603577	18.71439	#VALUE!	0.000103	30.90821	0.035899
blauwe bessen	2901-1	137.2167	23.11545	3.083917	3.984082	0.003913	0.152674	73.77097	0.004216	0.000209	31.72928	0.004132
blauwe bessen	2901-2	147.7995	26.33564	2.453146	5.100946	#VALUE!	0.17722	82.38963	0.004836	0	39.73806	0.021381
blauwe bessen	2901-3	118.4583	21.30872	2.453623	4.201435	#VALUE!	0.170831	70.35062	0.006819	0	30.53834	0.012835
druiven	2894-1	37.75798	13.79786	0.476807	13.43014	0.016764	0.373174	1.585265	0.099944	0.000134	16.50186	0.798723
druiven	2894-2	35.97381	13.18823	1.594544	13.68484	0.017904	0.849283	0.616142	0.220951	0	16.41534	0.580881
druiven	2894-3	34.67729	13.50195	0.577525	14.00449	0.010293	0.433249	0.3555	0.10245	0	17.76442	0.667682
framboos	2898-1	0.32368	28.15033	0.3106	0.051756	0.005715	7.345866	0.151101	0.039121	0.000224	1.937212	0.0129
framboos	2898-2	0.248459	31.55578	0.207539	0.05341	0.004348	5.629571	0.127531	0.021405	0.000158	1.877613	0.002805
framboos	2898-3	0.225853	30.90682	0.165536	0.054854	0.004717	4.604926	0.121127	0.016256	7.61E-05	1.456985	0.01271
bloemkool	2900-1	0.096023	0.499871	0.010051	0.003095	#VALUE!	0.017288	0.10829	#VALUE!	0.347189	0.017516	0.006134
bloemkool	2900-2	0.108327	0.235283	0.007757	0.003369	#VALUE!	0.00788	0.106168	#VALUE!	0.382364	0.011522	0.006117
bloemkool	2900-3	0.074634	0.162574	0.005473	0.002871	#VALUE!	0.00661	0.098846	#VALUE!	0.379373	0.009149	0.003188
spruiten	2893-1	#VALUE!	0.152823	0.003486	0.001623	#VALUE!	0.00701	3.424832	0.005319	0.005231	0.008838	0.014519
spruiten	2893-2	0.00964	0.128322	0.005553	0.001703	#VALUE!	0.0022	3.079798	0.003027	0.005475	0.007562	0.009952

spruiten	2893-3	0	0.104122	0.004207	0.001222	#VALUE!	0.003731	3.808507	0.010954	0.007364	0.009717	0.008159
tomaat	2896-1	0.05609	0.104793	0.006932	0.001382	#VALUE!	0.002475	11.35355	0.009944	6.61E-05	0.58357	0.023997
tomaat	2896-2	0.010613	0.031223	0.005268	0.000821	#VALUE!	#VALUE!	13.07515	0	0.000114	0.580531	0.024688
tomaat	2896-3	0.007211	0.030007	0.007978	0.000656	#VALUE!	0.003084	13.00476	0	7.34E-05	0.767166	0.022411
broccoli	2897-1	#VALUE!	0.022137	0.002948	0.000477	#VALUE!	0.002121	2.353087	#VALUE!	0.085389	0.06682	0.007609
broccoli	2897-2	#VALUE!	0.045901	0.002097	0.000506	#VALUE!	0.001232	2.066943	0.001721	0.0797	0.032161	0.006784
broccoli	2897-3	#VALUE!	0.047726	0.001626	0.000449	#VALUE!	0.00148	1.722623	#VALUE!	0.073134	0.043682	0.007618
wortel	2903-1	0.00428	0.021466	0.001609	0.000268	#VALUE!	0.002447	31.67324	0	0.000172	0.00259	0.005364
wortel	2903-2	0.005466	0.02242	0.002856	0.000229	#VALUE!	0.001322	33.3936	0	0.000199	0.001728	0.018815
wortel	2903-3	0.004684	0.019664	0.002978	0.000318	#VALUE!	0.001167	30.30897	0	0.000241	0.001806	0.003563
paprika	2902-1	0.005938	0.065973	0.002235	0.00042	#VALUE!	0.011374	0.994171	0.002994	0.000318	0.135384	0.326104
paprika	2902-2	0.006848	0.068685	0.005048	0.000415	#VALUE!	0.013461	0.436657	0.00268	0.000514	0.144785	0.340414
paprika	2902-3	0.005961	0.06661	0.003117	0.00045	#VALUE!	0.013187	0.203186	0	0.000669	0.142342	0.304559
ECP naturel 1	3126-1	0.014627	0.119331	0.17741	0.002142	#VALUE!	0.037003	10.050	#VALUE!	0.005748	0.009991	0.104291
ECP naturel 1	3126-2	#VALUE!	0.136538	0.186692	0.001805	#VALUE!	0.034314	10.361	#VALUE!	0.002932	0.011861	0.120861
ECP naturel 1	3126-3	0.035875	0.160784	0.161513	0.001508	#VALUE!	0.031048	9.416	#VALUE!	0.002807	0.00787	0.12204
ECP VFMX 100 - 400 06-07-22 1	3128-1	7.346422	69.12734	2.486538	2.05853	9.112052	6.779136	28.737	2.326137	3.0115	14.68286	0.232194
ECP VFMX 100 - 400 06-07-22 2	3128-2	7.342972	69.35131	2.269881	1.993943	9.689073	6.149262	29.595	2.450683	2.865128	14.66676	0.132163
ECP VFMX 100 - 400 06-07-22 3	3128-3	7.934349	80.11364	2.740699	2.419714	10.58852	8.485271	30.636	2.668667	3.220981	16.05532	0.268645
Mifood blends 2019 1	2905-1	0.014772	0.128516	0.725845	0.004535	0.035103	3.090928	14.85357	0.01325	0.000477	3.605711	0.026008
Mifood blends 2019 1	2905-2	0.004328	0.047342	0.611758	0.002829	0.016067	2.613037	14.48907	0.007505	0.009038	3.61274	0.016596

Mifood blends 2019 1	2905-3	0.006143	0.046155	0.633391	0.005257	0.013244	2.775351	14.54317	0.008438	0.100491	3.814797	0.024508
Mifood blends 2019 2	2906-1	53.5113	107.573	11.23849	12.52929	0.040388	6.975248	12.58806	0.873125	0.001983	18.4948	0.448377
Mifood blends 2019 2	2906-2	46.82427	100.0025	10.70873	11.6231	0.056759	6.889941	12.23124	0.845299	0.002065	17.77864	0.463638
Mifood blends 2019 2	2906-3	52.0294	109.8084	11.13362	12.54659	0.059567	6.78798	12.58056	0.826842	0.003184	18.23011	0.440301
Mifood blends 2019 3	2907-1	0.315924	1.735295	0.066572	0.032591	#VALUE!	0.03342	23.46223	0.011198	5.1E-05	0.390731	0.074757
Mifood blends 2019 3	2907-2	0.107738	0.486793	0.02007	0.007542	0.005998	0.012964	24.14099	0.00687	0.003997	0.334595	0.084643
Mifood blends 2019 3	2907-3	0.068975	0.283564	0.013601	0.006443	0.003642	0.008546	24.21738	0.010646	0.010064	0.329009	0.095954
Mifood blends 2019 4	2908-1	0.065506	0.15088	0.008735	0.003892	#VALUE!	0.004993	0.828186	0.003003	1.747357	0.023629	0.011754
Mifood blends 2019 4	2908-2	0.042909	0.127713	0.005519	0.003217	#VALUE!	0.005625	0.359783	#VALUE!	1.923086	0.026489	0.026159
Mifood blends 2019 4	2908-3	0.032004	0.085564	0.006732	0.002478	#VALUE!	0.005585	0.473465	#VALUE!	2.052813	0.025013	0.039887
Mifood blends 2019 5	2909-1	25.26774	78.40439	9.215161	8.735965	2.858845	7.292129	24.60394	1.512008	0.00037	15.32951	0.276226
Mifood blends 2019 5	2909-2	23.07466	73.9193	9.811287	8.065417	2.722722	7.780573	23.49195	1.418413	0.000547	13.83596	0.215559
Mifood blends 2019 5	2909-3	22.46387	75.86414	9.353112	8.539997	2.737505	6.427243	21.09856	1.410649	0.000236	14.6384	0.268385
Mifood blends 2019 6	2910-1	5.302479	49.89468	10.71177	4.813484	13.46077	8.264056	32.19544	2.571836	9.72E-05	6.971702	0.28516
Mifood blends 2019 6	2910-2	5.238626	48.2297	10.55184	4.858512	14.10791	8.515671	33.00674	2.645304	0.000225	7.240198	0.289877
Mifood blends 2019 6	2910-3	5.153229	47.12283	10.66315	4.692166	13.5828	8.622869	32.80399	2.700363	0.000155	7.065725	0.26755
Mifood blends 2019 7	2911-1	1.634026	18.75415	3.353101	1.998876	3.598547	1.922971	6.93281	0.754145	5.226247	3.288244	0.135418
Mifood blends 2019 7	2911-2	1.69897	19.09186	3.055355	2.086684	3.572007	1.743332	6.955683	0.654453	5.532709	3.293329	0.12645
Mifood blends 2019 7	2911-3	1.509714	17.48071	2.708462	1.891977	3.297314	1.542005	6.806828	0.62001	5.016554	3.235585	0.120632
thee	2904-1	0.068659	0.557801	23.25638	0.008065	366.8658	58.07141	5.731364	84.16012	0.005114	18.37257	0.014045
thee	2904-2	0.043554	0.273924	22.48881	0.004299	358.1672	57.36741	5.891049	83.74353	0.001218	18.17909	0.023075

thee	2904-3	0.037359	0.221915	23.03374	0.003771	366.4723	57.38584	5.826125	85.54436	0.020221	18.57579	0.021502
t 1 month												
		**[mg/kg]**										
**Naam**	**BL-nummer**	**Delphinidin-3-glucoside**	**Cyanidin-3-glucoside**	**Catechin**	**Peonidin-3-glucoside**	**Epigallocatechin-3-gallate**	**Epicatechin**	**Chlorogenic acid**	**Epicatechin gallate**	**Sulforaphane**	**Quercetin-3-glucoside**	**Resveratrol**
Pas-v 1	BL-2951-1	6.510	56.277	0.698	2.565	2.356	2.603	7.641	1.721	2.497	6.818	0.048
Pas-v 1	BL-2951-2	4.361	40.741	0.534	1.978	2.064	2.156	6.472	1.368	2.017	5.231	0.020
Pas-v 1	BL-2951-3	5.611	51.056	0.661	2.407	2.227	2.544	7.139	1.640	2.316	6.495	0.039
o-v 1	BL-2943-1	6.295	51.197	0.974	2.126	3.001	4.285	12.304	1.642	2.327	6.248	0.071
o-v 1	BL-2943-2	6.282	52.596	1.025	2.531	3.217	4.846	13.199	1.878	2.397	6.253	0.043
o-v 1	BL-2943-3	5.581	42.833	0.912	2.270	2.869	4.181	12.322	1.609	2.344	5.958	0.051
Pst-v 1	BL-2939-1	3.435	33.214	1.336	1.478	3.265	7.943	17.177	1.578	1.634	4.735	0.048
Pst-v 1	BL-2939-2	4.095	35.152	1.440	1.695	3.514	8.755	18.219	1.588	1.771	5.076	0.055
Pst-v 1	BL-2939-3	3.750	34.283	1.347	1.530	3.226	7.743	17.287	1.486	1.545	4.867	0.061
ECP VFMX 100 - 400 06-07-22 3	BL-3130-1	2.985	47.367	1.988	2.010	5.001	7.858	42.866	2.821	2.279	16.015	*
ECP VFMX 100 - 400 06-07-22 3	BL-3130-2	3.283	47.626	1.864	1.880	4.888	6.672	38.011	2.785	1.944	14.704	*
ECP VFMX 100 - 400 06-07-22 3	BL-3130-3	2.741	42.847	1.699	1.781	4.132	6.040	34.023	2.224	1.955	13.535	*
t 6 month												
**Naam**	**BL-nummer**	**Delphinidin-3-glucoside**	**Cyanidin-3-glucoside**	**Catechin**	**Peonidin-3-glucoside**	**Epigallocatechin-3-gallate**	**Epicatechin**	**Chlorogenic acid**	**Epicatechin gallate**	**Sulforaphane**	**Quercetin-3-glucoside**	**Resveratrol**
Pst-v-3	BL-2022-2941-1	1.99	13.67	0.83	0.66	3.72	3.64	15.96	1.14	0.80	4.73	0.04
Pst-v-3	BL-2022-2941-2	2.03	15.72	0.86	0.67	3.91	3.70	16.42	1.16	0.83	5.28	0.05
Pst-v-3	BL-2022-2941-3	2.15	16.44	0.89	0.63	3.79	3.55	15.84	1.19	0.75	4.52	0.04
O-v-3	BL-2022-2945-1	1.48	17.99	0.35	0.78	1.54	1.10	5.10	0.79	0.92	4.63	0.02

O-v-3	BL-2022-2945-2	1.56	19.55	0.40	0.89	1.52	1.15	5.44	0.83	1.04	4.74	0.02
O-v-3	BL-2022-2945-3	1.51	18.41	0.32	0.77	1.37	0.93	4.55	0.81	0.94	5.07	0.03
Pas-v-3	BL-2022-2953-1	1.27	17.78	0.29	0.80	1.03	0.87	2.62	0.74	0.97	5.04	0.01
Pas-v-3	BL-2022-2953-2	0.89	14.38	0.27	0.64	0.81	0.79	2.14	0.59	0.88	4.51	0.02
Pas-v-3	BL-2022-2953-3	1.19	17.52	0.33	0.78	1.10	0.94	2.71	0.76	0.96	5.48	0.00
ECP- VFMX 06-07-22 2	BL-2022-3129-1	1.80	6.79	0.63	0.26	4.07	1.52	28.53	1.43	0.59	12.55	0.08
ECP- VFMX 06-07-22 2	BL-2022-3129-2	1.34	6.10	0.65	0.27	4.01	1.73	27.98	1.47	0.60	12.06	0.06
ECP- VFMX 06-07-22 2	BL-2022-3129-3	1.31	5.60	0.57	0.23	3.77	1.41	24.38	1.26	0.54	11.85	0.12

A2: Polyphenol RSDs

t0		RSD										
Naam	BL-nummer	Delphinidin-3-glucoside	Cyanidin-3-glucoside	Catechin	Peonidin-3-glucoside	Epigallocatechin-3-gallate	Epicatechin	Chlorogenic acid	Epicatechin gallate	Sulforaphane	Quercetin-3-glucoside	Resveratrol
Pas-k	2950-1	1.790541	3.20538	5.330254	3.780758	2.551836	9.031946	3.13187	1.496238	0.753588	0.89621	17.82209
Pas-k	2950-2											
Pas-k	2950-3											
Pst-k	2938-1	6.206752	6.130172	7.541	2.586513	2.465894	6.164371	4.145112	5.052792	3.342855	3.428131	2.198532
Pst-k	2938-2											
Pst-k	2938-3											
o-k	2942-1	1.104662	1.112019	1.96122	4.769493	1.871791	5.990796	2.752119	3.156955	4.156058	3.451018	65.12626
o-k	2942-2											
o-k	2942-3											
Pas-v 1	2951-1	5.475591	4.061941	5.245651	6.040308	1.532943	7.727658	1.631163	3.375893	1.331792	2.548286	43.85083
Pas-v 1	2951-2											
Pas-v 1	2951-3											
Pst-v 1	2939-1	3.610857	2.184537	3.311686	0.580622	0.275255	3.39357	1.77411	1.096797	1.094461	4.626455	6.935979
Pst-v 1	2939-2											

Pst-v 1	2939-3											
o-v 1	2943-1	22.08384	15.70819	13.87847	11.15118	17.85752	19.49483	11.08907	6.362513	7.850409	7.727668	18.77892
o-v 1	2943-2											
o-v 1	2943-3											
bramen	2899-1	61.6956	5.481937	7.617449	7.833162	67.02805	5.517814	22.7772	23.52095	43.02913	11.45136	35.56372
bramen	2899-2											
bramen	2899-3											
appel	2895-1	26.41486	8.236309	11.20372	10.73902	42.53608	9.410355	9.958685	#VALUE!	173.2051	6.467261	46.08953
appel	2895-2											
appel	2895-3											
blauwe bessen	2901-1	11.04842	10.7958	13.66733	13.37005	#VALUE!	7.629602	8.216463	25.70128	173.2051	14.71455	67.47262
blauwe bessen	2901-2											
blauwe bessen	2901-3											
druiven	2894-1	4.280347	2.258876	70.02653	2.099669	27.39013	46.98037	76.02986	49.00365	173.2051	4.470007	16.07012
druiven	2894-2											
druiven	2894-3											
framboos	2898-1	19.25482	5.98639	32.75352	2.906396	14.3544	23.63326	11.84599	46.86533	48.45965	14.89563	60.96636
framboos	2898-2											
framboos	2898-3											
bloemkool	2900-1	18.33362	59.32036	29.49964	8.029258	#VALUE!	55.0657	4.744672	#VALUE!	5.276074	33.87544	32.95703
bloemkool	2900-2											
bloemkool	2900-3											
spruiten	2893-1	#VALUE!	18.96159	23.76466	16.98768	#VALUE!	56.97378	10.60372	63.40634	19.38604	12.44773	30.15227
spruiten	2893-2											
spruiten	2893-3											
tomaat	2896-1	110.7678	77.38883	20.31788	39.90976	#VALUE!	#VALUE!	7.808101	173.2051	30.51978	16.60372	4.926729
tomaat	2896-2											
tomaat	2896-3											

broccoli	2897-1	#VALUE!	36.99703	30.12922	5.951338	#VALUE!	28.47899	15.4174	#VALUE!	7.723316	37.11786	6.527222
broccoli	2897-2											
broccoli	2897-3											
wortel	2903-1	12.53318	6.606359	30.54827	16.43757	#VALUE!	42.45884	4.862033	#DIV/0!	17.11656	23.36409	90.12715
wortel	2903-2											
wortel	2903-3											
paprika	2902-1	8.305374	2.113897	41.48853	4.444166	#VALUE!	8.950215	74.61463	87.00004	35.11658	3.463525	5.575883
paprika	2902-2											
paprika	2902-3											
ECP naturel 1	3126-1	#VALUE!	14.99499	7.267625	17.43874	#VALUE!	8.738727	4.843758	#VALUE!	43.43314	20.15651	8.57573
ECP naturel 1	3126-2											
ECP naturel 1	3126-3											
ECP VFMX 100 - 400 06-07-22 1	3128-1	4.514375	8.617806	9.429937	10.63587	7.595288	16.93246	3.207333	6.985826	5.897922	5.26644	33.49094
ECP VFMX 100 - 400 06-07-22 2	3128-2											
ECP VFMX 100 - 400 06-07-22 3	3128-3											
Mifood blends 2019 1	2905-1	66.32155	63.79645	9.223232	29.63174	55.37359	8.597629	1.34461	31.67973	151.1856	3.228569	22.60538
Mifood blends 2019 1	2905-2											
Mifood blends 2019 1	2905-3											
Mifood blends 2019 2	2906-1	6.915019	4.857684	2.544028	4.318262	19.82881	1.361884	1.635392	2.746142	27.82497	1.99319	2.629131
Mifood blends 2019 2	2906-2											
Mifood blends 2019 2	2906-3											
Mifood blends 2019 3	2907-1	80.87594	94.11749	86.48028	95.26206	#VALUE!	72.47678	1.736388	24.61357	107.2282	9.713331	12.46096
Mifood blends 2019 3	2907-2											
Mifood blends 2019 3	2907-3											

Mifood blends 2019 4	2908-1	36.50741	27.28044	23.21918	22.13499	#VALUE!	6.550851	44.11601	#VALUE!	8.03586	5.71175	54.24725
Mifood blends 2019 4	2908-2											
Mifood blends 2019 4	2908-3											
Mifood blends 2019 5	2909-1	6.247196	2.956936	3.298876	4.081696	2.693445	9.562841	7.766373	3.898478	40.65652	5.119173	13.0219
Mifood blends 2019 5	2909-2											
Mifood blends 2019 5	2909-3											
Mifood blends 2019 6	2910-1	1.431415	2.88183	0.770372	1.796953	2.506746	2.175206	1.292437	2.443298	40.17584	1.920922	4.189836
Mifood blends 2019 6	2910-2											
Mifood blends 2019 6	2910-3											
Mifood blends 2019 7	2911-1	5.957448	4.607117	10.61647	4.893786	4.779896	10.97778	1.16198	10.30197	4.936483	0.977014	5.842209
Mifood blends 2019 7	2911-2											
Mifood blends 2019 7	2911-3											
thee	2904-1	33.24431	51.4759	1.722485	43.53834	1.350204	0.696498	1.380727	1.11591	113.4133	1.079517	24.68578
thee	2904-2											
thee	2904-3											
t 1 month												
		RSD										
**Naam**	**BL-nummer**	Delphinidin-3-glucoside	Cyanidin-3-glucoside	Catechin	Peonidin-3-glucoside	Epigallocatechin-3-gallate	Epicatechin	Chlorogenic acid	Epicatechin gallate	Sulforaphane	Quercetin-3-glucoside	Resveratrol
Pas-v 1	BL-2951-1	19.64586	16.0184	13.56977	13.10147	6.597867	9.97206	8.281622	11.7213	10.64631	13.56609	39.79768
Pas-v 1	BL-2951-2											
Pas-v 1	BL-2951-3											

o-v 1	BL-2943-1	6.744934	10.80161	5.788136	8.893837	5.80677	8.065794	4.056281	8.573357	1.550583	2.747803	26.41347
o-v 1	BL-2943-2											
o-v 1	BL-2943-3											
Pst-v 1	BL-2939-1	8.777326	2.837391	4.159461	7.211908	4.693282	6.578302	3.260114	3.627929	6.894107	3.508802	11.70087
Pst-v 1	BL-2939-2											
Pst-v 1	BL-2939-3											
ECP VFMX 100 - 400 06-07-22 3	BL-3130-1	9.039606	5.849626	7.830897	6.06429	10.10909	13.46336	11.56286	12.8321	9.245611	8.409185	#DIV/0!
ECP VFMX 100 - 400 06-07-22 3	BL-3130-2											
ECP VFMX 100 - 400 06-07-22 3	BL-3130-3											
t 6 month												
**Naam**	**BL-nummer**	Delphinidin-3-glucoside	Cyanidin-3-glucoside	Catechin	Peonidin-3-glucoside	Epigallocatechin-3-gallate	Epicatechin	Chlorogenic acid	Epicatechin gallate	Sulforaphane	Quercetin-3-glucoside	Resveratrol
Pst-v-3	BL-2022-2941-1	3.997193	9.40135	3.771704	2.84804	2.546816	2.062486	1.891382	2.037378	4.731611	8.135343	9.235959
Pst-v-3	BL-2022-2941-2											
Pst-v-3	BL-2022-2941-3											
O-v-3	BL-2022-2945-1	2.491953	4.319615	11.35692	8.070359	6.214475	10.98258	8.882688	2.026606	6.439884	4.744822	25.38903
O-v-3	BL-2022-2945-2											
O-v-3	BL-2022-2945-3											

Pas-v-3	BL-2022-2953-1	17.71378	11.42049	8.984161	11.92709	15.35746	8.694471	12.26907	13.17322	4.976667	9.709338	85.06079
Pas-v-3	BL-2022-2953-2											
Pas-v-3	BL-2022-2953-3											
ECP- VFMX 06-07-22 2	BL-2022-3129-1	18.71941	9.773372	6.470998	8.65833	4.068466	10.46936	8.365133	8.078934	5.855386	2.969671	36.61015
ECP- VFMX 06-07-22 2	BL-2022-3129-2											
ECP- VFMX 06-07-22 2	BL-2022-3129-3											

B: Vitamin C Measurements and RSD

**Naam**	**BL-nummer**	**Concentration [mg/kg]**	average	RSD
ECP VFMX 100 - 400 06-07-22 1	BL-2022-3128-1	#VALUE!	#VALUE!	#VALUE!
ECP VFMX 100 - 400 06-07-22 1	BL-2022-3128-2	#VALUE!		
Tomaat	BL-2022-4493-1	288.9834351	291.3527905	1.1500746
Tomaat	BL-2022-4493-2	293.7221458		
Bloemkool	BL-2022-4494-1	219.0262187	204.1178323	10.329153
Bloemkool	BL-2022-4494-2	189.2094459		
Blauwe bes	BL-2022-4495-1	16.63693443	17.16372211	4.3404937
Blauwe bes	BL-2022-4495-2	17.69050978		
Framboos	BL-2022-4496-1	198.5954354	197.785225	0.5793205
Framboos	BL-2022-4496-2	196.9750147		
Rode paprika	BL-2022-4497-1	1092.284726	1090.313592	0.2556699
Rode paprika	BL-2022-4497-2	1088.342459		
Appel	BL-2022-4498-1	40.41510956	41.30216555	3.0373386
Appel	BL-2022-4498-2	42.18922154		
Wortel	BL-2022-4499-1	23.31873454	21.48664722	12.058478
Wortel	BL-2022-4499-2	19.65455991		

Broccoli	BL-2022-4500-1	529.7972714	500.6651109	8.2288731
Broccoli	BL-2022-4500-2	471.5329504		
Braam	BL-2022-4501-1	42.76776835	43.33491508	1.8508554
Braam	BL-2022-4501-2	43.90206181		
Thee	BL-2022-4502-1	#VALUE!	#VALUE!	#VALUE!
Thee	BL-2022-4502-2	#VALUE!		
Blauwe druif	BL-2022-4503-1	19.92029851	19.62691797	2.1139475
Blauwe druif	BL-2022-4503-2	19.33353744		
Spruit	BL-2022-4504-1	569.4562978	551.1714081	4.6915966
Spruit	BL-2022-4504-2	532.8865183		
Pasc-k	BL-2022-4573-1	84.10812907	83.918188	0.3200942
Pasc-k	BL-2022-4573-2	83.72824692		
Pasc-v	BL-2022-4574-1	129.8132394	129.9350831	0.1326147
Pasc-v	BL-2022-4574-2	130.0569268		
O-k	BL-2022-4575-1	79.35471265	84.2063742	8.1481784
O-k	BL-2022-4575-2	89.05803574		
O-v	BL-2022-4576-1	143.4880627	143.3728058	0.1136881
O-v	BL-2022-4576-2	143.2575489		
Pasteur-k	BL-2022-4577-1	51.06788074	51.34569727	0.7651895
Pasteur-k	BL-2022-4577-2	51.6235138		
Pasteur-v	BL-2022-4578-1	70.47004468	66.30334285	8.8873441
Pasteur-v	BL-2022-4578-2	62.13664101		
t1 month				
**Naam**	**BL-nummer**	**L- ascorbic acid**	average	RSD
ECP VFMX 100 - 400 06-07-22 1	BL-2022-3128-1		#DIV/0!	#DIV/0!
ECP VFMX 100 - 400 06-07-22 1	BL-2022-3128-2			
Pas-v	BL-2022-2951-1	70.537	72.595	4.009
Pas-v	BL-2022-2951-2	74.653		
O-v	BL-2022-2943-1		#DIV/0!	#DIV/0!
O-v	BL-2022-2943-2			

Pst-v	BL-2022-2939-1	0	0.000	#DIV/0!
Pst-v	BL-2022-2939-2	0		
t6 month				
		**[mg/kg]**		
**Naam**	**BL-nummer**	**L- ascorbic acid**	average	RSD
Pst-v-3	BL-2022-2941-1	18.3	18.3	1.6475254
Pst-v-3	BL-2022-2941-2	18.1		
Pst-v-3	BL-2022-2941-3	18.7		
O-v-3	BL-2022-2945-1	56.5	56.7	0.3581227
O-v-3	BL-2022-2945-2	56.9		
O-v-3	BL-2022-2945-3	56.6		
Pas-v-3	BL-2022-2953-1	44.8	45.0	0.5177849
Pas-v-3	BL-2022-2953-2	45.2		
Pas-v-3	BL-2022-2953-3	45.1		
ECP- VFMX 06-07-22 2	BL-2022-3129-1	-	#DIV/0!	#DIV/0!
ECP- VFMX 06-07-22 2	BL-2022-3129-2	-		
ECP- VFMX 06-07-22 2	BL-2022-3129-3	-		
t1 month		**[mg/kg]**		
**Naam**	**BL-nummer**	**L- ascorbic acid**	average	RSD
Bloemkool	BL-2022-2900-1	274.084	256.0	6.1283688
Bloemkool	BL-2022-2900-2	247.787		
Bloemkool	BL-2022-2900-3	246.112		
Spruiten	BL-2022-2893-1	528.934	493.3	10.335725
Spruiten	BL-2022-2893-2	516.100		
Spruiten	BL-2022-2893-3	434.906		
Tomaat	BL-2022-2896-1	125.285	160.8	23.16411
Tomaat	BL-2022-2896-2	157.441		
Tomaat	BL-2022-2896-3	199.539		
Wortel	BL-2022-2903-1	18.041	17.3	10.188985

Wortel	BL-2022-2903-2	15.333		
Wortel	BL-2022-2903-3	18.654		
Paprika	BL-2022-2902-1	804.446	874.2	14.773864
Paprika	BL-2022-2902-2	794.981		
Paprika	BL-2022-2902-3	1023.272		
ECP naturel 1	BL-2022-3126-1	*	#DIV/0!	#DIV/0!
ECP naturel 1	BL-2022-3126-2	*		
ECP naturel 1	BL-2022-3126-3	*		
Thee	BL-2022-2904-1	*	#DIV/0!	#DIV/0!
Thee	BL-2022-2904-2	*		
Thee	BL-2022-2904-3	*		
Mifood blends 2019 1	BL-2022-2905-1	1.045	0.9	17.533965
Mifood blends 2019 1	BL-2022-2905-2	0.743		
Mifood blends 2019 1	BL-2022-2905-3	0.843		
Mifood blends 2019 2	BL-2022-2906-1	0.793	0.8	9.1977885
Mifood blends 2019 2	BL-2022-2906-2	0.805		
Mifood blends 2019 2	BL-2022-2906-3	0.933		
Mifood blends 2019 3	BL-2022-2907-1	88.585	89.1	0.8112303
Mifood blends 2019 3	BL-2022-2907-2	89.909		
Mifood blends 2019 3	BL-2022-2907-3	88.745		
Mifood blends 2019 4	BL-2022-2908-1	*	#DIV/0!	#DIV/0!
Mifood blends 2019 4	BL-2022-2908-2	*		
Mifood blends 2019 4	BL-2022-2908-3	*		
Mifood blends 2019 5	BL-2022-2909-1	0.923	0.8	16.592852
Mifood blends 2019 5	BL-2022-2909-2	0.796		
Mifood blends 2019 5	BL-2022-2909-3	0.660		
Mifood blends 2019 6	BL-2022-2910-1	1.807	1.8	1.9149749
Mifood blends 2019 6	BL-2022-2910-2	1.746		
Mifood blends 2019 6	BL-2022-2910-3	1.804		
Mifood blends 2019 7	BL-2022-2911-1	1.549	1.5	3.285027
Mifood blends 2019 7	BL-2022-2911-2	1.456		
Mifood blends 2019 7	BL-2022-2911-3	1.532		
Druiven	BL-2894-1	36.66259969	36.98064732	1.2162774

Druiven	BL-2894-2	37.29869495		
Broccoli	BL-2897-1	480.4270554	500.0405846	5.5470936
Broccoli	BL-2897-2	519.6541138		
Pas v.1	BL-2951-1	70.53674767	72.5946643	4.0090186
Pas v.1	BL-2951-2	74.65258092		
ECP VFMX 100 - 400 06-07-22 3	BL-3130-1	#VALUE!	#VALUE!	#VALUE!
ECP VFMX 100 - 400 06-07-22 3	BL-3130-2	#VALUE!		
Pst-v 1	BL-2022-2939-1	13.790	13.2	6.2105877
Pst-v 1	BL-2022-2939-2	12.260		
Pst-v 1	BL-2022-2939-3	13.532		
o-v 1	BL-2022-2943-1	79.433	81.2	2.0888705
o-v 1	BL-2022-2943-2	82.822		
o-v 1	BL-2022-2943-3	81.207		
bramen	BL-2022-2899-1	69.459	71.9	3.2321822
bramen	BL-2022-2899-2	72.025		
bramen	BL-2022-2899-3	74.095		
appel	BL-2022-2895-1	57.479	68.8	21.521363
appel	BL-2022-2895-2	63.323		
appel	BL-2022-2895-3	85.535		
blauwe bessen	BL-2022-2901-1	2.305	2.2	7.5058846
blauwe bessen	BL-2022-2901-2	2.251		
blauwe bessen	BL-2022-2901-3	1.998		
framboos	BL-2022-2898-1	223.378	216.7	3.0661074
framboos	BL-2022-2898-2	210.095		
framboos	BL-2022-2898-3	216.496		
t6 month				
		**[mg/kg]**		
**Naam**	**BL-nummer**	**L- ascorbic acid**	average	RSD
Pst-v-3	BL-2022-2941-1	18.3	18.3	1.6475254
Pst-v-3	BL-2022-2941-2	18.1		
Pst-v-3	BL-2022-2941-3	18.7		

O-v-3	BL-2022-2945-1	56.5	56.7	0.3581227
O-v-3	BL-2022-2945-2	56.9		
O-v-3	BL-2022-2945-3	56.6		
Pas-v-3	BL-2022-2953-1	44.8	45.0	0.5177849
Pas-v-3	BL-2022-2953-2	45.2		
Pas-v-3	BL-2022-2953-3	45.1		
ECP- VFMX 06-07-22 2	BL-2022-3129-1	-	#DIV/0!	#DIV/0!
ECP- VFMX 06-07-22 2	BL-2022-3129-2	-		
ECP- VFMX 06-07-22 2	BL-2022-3129-3	-		

C: Carotenoid Measurements and RSDs

		mg/kg				RSD (%)			
code MiFood	BL-2022-	luteine	lyco	a-car	B-car	luteine	lyco	a-car	B-car
Pas-k	2950-1	1.441481	1.204535	2.751485	7.808797	23.17929929	27.24838	13.71403	20.10836
Pas-k	2950-2	2.130088	0.696547	2.115558	5.208101				
Pas-k	2950-3	1.470179	0.908242	2.297512	6.404042				
Pst-k	2938-1	2.695666	3.389606	4.003223	11.46692	9.836403328	33.80096	21.68395	20.65715
Pst-k	2938-2	2.794583	1.659045	4.157043	11.69939				
Pst-k	2938-3	3.233063	2.983514	5.826036	16.28383				
o-k	2942-1	1.879894	2.172697	4.571747	12.56211	7.913526556	9.011778	11.69622	10.13586
o-k	2942-2	2.20335	1.970819	3.722232	10.4527				
o-k	2942-3	2.052205	1.815696	3.793447	10.75384				
Pas-v 2	2952-1	2.438816	1.658053	3.377061	9.574644	12.226227	13.33006	13.19899	13.93922
Pas-v 2	2952-2	1.920266	1.831299	2.601077	7.258135				
Pas-v 2	2952-3	2.324027	1.399623	3.176257	8.944529				
Pst-v 2	2940-1	2.078022	1.656168	4.368574	12.29359	20.6006707	37.66145	35.54214	36.45497
Pst-v 2	2940-2	1.588407	0.789996	2.10377	5.793894				
Pst-v 2	2940-3	1.404327	1.737846	4.219194	11.89769				
o-v 2	2944-1	1.88461	1.888332	3.834788	10.54719	5.310104674	17.15025	14.18423	13.11735
o-v 2	2944-2	2.078192	1.683439	3.400436	9.428969				
o-v 2	2944-3	2.058693	2.34414	4.502157	12.22636				
ECP naturel 1	3126-1	#VALUE!	#VALUE!	#VALUE!	#VALUE!	#VALUE!	#VALUE!	#VALUE!	#VALUE!

ECP naturel 1	3126-2	#VALUE!	#VALUE!	#VALUE!	#VALUE!				
ECP naturel 1	3126-3	#VALUE!	#VALUE!	#VALUE!	#VALUE!				
ECP VFMX 100 - 400 06-07-22 1	3128-1	#VALUE!	#VALUE!	2.755734	7.387646	#VALUE!	#VALUE!	64.51332	32.84797
ECP VFMX 100 - 400 06-07-22 1	3128-2	#VALUE!	#VALUE!	2.666077	10.58685				
ECP VFMX 100 - 400 06-07-22 1	3128-3	#VALUE!	#VALUE!	7.537034	14.48423				
ECP VFMX 100 - 400 06-07-22 3	3130-1	#VALUE!	#VALUE!	4.446816	9.163343	#VALUE!	#VALUE!	25.36575	27.41574
ECP VFMX 100 - 400 06-07-22 3	3130-2	#VALUE!	#VALUE!	6.863538	15.6249				
ECP VFMX 100 - 400 06-07-22 3	3130-3	#VALUE!	#VALUE!	4.623333	11.27053				
Mifood blends 2019 3	2907-1	2.155622	2.788301	4.918638	18.06062	30.93103062	1.590067	5.099228	4.415448
Mifood blends 2019 3	2907-2	1.489381	2.810323	5.437172	19.42012				
Mifood blends 2019 3	2907-3	1.182058	2.725314	5.1038	19.5953				
Mifood blends 2019 4	2908-1	3.336246	#VALUE!	#VALUE!	1.076262	14.56208814	#VALUE!	#VALUE!	6.295846
Mifood blends 2019 4	2908-2	4.344064	#VALUE!	#VALUE!	1.134233				
Mifood blends 2019 4	2908-3	3.492812	#VALUE!	#VALUE!	1.219365				
Mifood blends 2019 6	2910-1	#VALUE!	3.1172	3.939569	14.21162	#VALUE!	37.87004	33.67521	32.77237
Mifood blends 2019 6	2910-2	#VALUE!	1.382088	2.007562	7.296697				
Mifood blends 2019 6	2910-3	#VALUE!	2.41466	2.741215	10.25236				
Mifood blends 2019 7	2911-1	2.770812	1.701984	2.341087	8.58202	9.519257269	9.620009	17.80019	4.791667
Mifood blends 2019 7	2911-2	2.459719	1.537788	1.647685	7.82433				
Mifood blends 2019 7	2911-3	2.977057	1.404661	2.205713	8.039489				
Bloemkool	2900-1	#VALUE!	#VALUE!	#VALUE!	#VALUE!	#VALUE!	#VALUE!	#VALUE!	#VALUE!
Bloemkool	2900-2	#VALUE!	#VALUE!	#VALUE!	#VALUE!				
Bloemkool	2900-3	#VALUE!	#VALUE!	#VALUE!	#VALUE!				
Spruiten	2893-1	5.868934	#VALUE!	#VALUE!	#VALUE!	18.36154089	#VALUE!	#VALUE!	#VALUE!
Spruiten	2893-2	7.928773	#VALUE!	#VALUE!	#VALUE!				
Spruiten	2893-3	5.828127	#VALUE!	#VALUE!	#VALUE!				
Tomaat	2896-1	2.426727	5.421946	#VALUE!	2.747887	5.051908915	11.3831	#VALUE!	4.135777
Tomaat	2896-2	2.466131	6.415905	#VALUE!	2.660093				
Tomaat	2896-3	2.664189	6.789367	#VALUE!	2.530356				
Broccoli	2897-1	13.69486	#VALUE!	#VALUE!	0.538146	28.40847997	#VALUE!	#VALUE!	28.21353
Broccoli	2897-2	11.48693	#VALUE!	#VALUE!	0.82624				

Broccoli	2897-3	7.570767	#VALUE!	#VALUE!	0.968911				
Wortel	2903-1	3.704258	#VALUE!	15.08299	31.8984	36.86466356	#VALUE!	39.33471	39.64366
Wortel	2903-2	2.308961	#VALUE!	6.493668	13.55699				
Wortel	2903-3	5.020194	#VALUE!	13.85874	28.72506				
Paprika	2902-1	3.190667	#VALUE!	#VALUE!	1.608821	14.63246495	#VALUE!	#VALUE!	15.10596
Paprika	2902-2	3.555231	#VALUE!	#VALUE!	1.54606				
Paprika	2902-3	4.246254	#VALUE!	#VALUE!	1.201417				
T= 1 maand									
	rc	mg/kg				RSD (%)			
**Naam**	**BL-nummer**	luteine	lyco	a-car	B-car	luteine	lyco	a-car	B-car
Pas-v 1	BL-2951-1	1.379509	2.007839	3.856197	13.37199	29.56846106	4.798909	6.765621	3.85979
Pas-v 1	BL-2951-2	2.04688	1.872342	3.588988	12.90814				
Pas-v 1	BL-2951-3	1.179964	1.833817	3.369193	12.37798				
o-v 1	BL-2943-1	1.402949	2.148094	4.057681	14.03312	31.88205483	13.04105	9.688155	6.626883
o-v 1	BL-2943-2	1.887068	2.304978	3.987949	14.67306				
o-v 1	BL-2943-3	2.656016	1.777524	3.386236	12.86235				
Pst-v 1	BL-2939-1	2.128907	2.082273	4.899648	17.83091	25.51151399	12.00246	4.232806	2.623595
Pst-v 1	BL-2939-2	2.162081	2.582435	5.332616	18.78525				
Pst-v 1	BL-2939-3	3.256849	2.587128	5.138612	18.41677				
ECP VFMX 100 - 400 06-07-22 3	BL-3130-1	#VALUE!	#VALUE!	4.443354	13.13518	#VALUE!	#VALUE!	6.043872	1.516627
ECP VFMX 100 - 400 06-07-22 3	BL-3130-2	#VALUE!	#VALUE!	4.846106	13.12778				
ECP VFMX 100 - 400 06-07-22 3	BL-3130-3	#VALUE!	#VALUE!	4.322328	12.78959				
t 6 month		c. (mg/kg)				rsd %			
**Naam**	**BL-nummer**	**B-car**	**a-car**	**lycopen e**	**lutein**	**B-car**	**a-car**	**lycopen e**	**lutein**
Pst-v-3	BL-2022-2941-1	15.17	5.14	5.25	3.19	3.615431989	2.526566	5.315825	2.130267
Pst-v-3	BL-2022-2941-2	15.13	4.90	5.49	3.29				
Pst-v-3	BL-2022-2941-3	14.22	4.95	4.93	3.16				
O-v-3	BL-2022-2945-1	12.38	4.56	4.26	2.94	3.757179639	3.224336	4.968803	2.032296
O-v-3	BL-2022-2945-2	11.58	4.71	3.89	3.06				

O-v-3	BL-2022-2945-3	11.63	4.42	3.95	3.02				
Pas-v-3	BL-2022-2953-1	10.86	4.31	3.69	2.78	9.260186575	7.85611	11.20139	1.680676
Pas-v-3	BL-2022-2953-2	9.14	3.70	2.96	2.85				
Pas-v-3	BL-2022-2953-3	9.48	3.88	3.25	2.76				
ECP- VFMX 06-07-22 2	BL-2022-3129-1	5.52	2.14	#VALUE!	#VALUE!	50.85831363	46.10885	#VALUE!	#VALUE!
ECP- VFMX 06-07-22 2	BL-2022-3129-2	2.50	0.92	#VALUE!	#VALUE!				
ECP- VFMX 06-07-22 2	BL-2022-3129-3	2.42	1.15	#VALUE!	#VALUE!				
